# Preventing autosomal-dominant hearing loss in *Bth* mice with CRISPR/CasRx-based RNA editing

**DOI:** 10.1038/s41392-022-00893-4

**Published:** 2022-03-14

**Authors:** Ziwen Zheng, Guo Li, Chong Cui, Fang Wang, Xiaohan Wang, Zhijiao Xu, Huiping Guo, Yuxin Chen, Honghai Tang, Daqi Wang, Mingqian Huang, Zheng-Yi Chen, Xingxu Huang, Huawei Li, Geng-Lin Li, Xiaoxiang Hu, Yilai Shu

**Affiliations:** 1 ENT Institute and Department of Otorhinolaryngology, Eye & ENT Hospital, State Key Laboratory of Medical Neurobiology and MOE Frontiers Center for Brain Science, Fudan University, Shanghai, 200031 China; 2 Institutes of Biomedical Sciences, Fudan University, Shanghai, 200032 China; 3 NHC Key Laboratory of Hearing Medicine (Fudan University), Shanghai, 200032 China; 4 State Key Laboratory of Agrobiotechnology, China Agricultural University, Beijing, 100193 China; 5 College of Biological Sciences, China Agricultural University, Beijing, 100193 China; 6 School of Life Science and Technology, Southeast University, Nanjing, 210096 China; 7 Department of Otolaryngology-Head and Neck Surgery, Graduate Program in Speech and Hearing Bioscience and Technology and Program in Neuroscience, Harvard Medical School, Boston, MA 02115 USA; 8 Eaton-Peabody Laboratory, Massachusetts Eye and Ear Infirmary, Boston, MA 02114 USA; 9 School of Life Science and Technology, ShanghaiTech University, Shanghai, 200031 China; 10 CAS Center for Excellence in Molecular Cell Science, Shanghai Institute of Biochemistry and Cell Biology, Chinese Academy of Sciences, University of Chinese Academy of Sciences, Shanghai, 200031 China; 11 Institutes of Brain Science and the Collaborative Innovation Center for Brain Science, Fudan University, Shanghai, 200031 China

**Keywords:** Neurodevelopmental disorders, Neurodevelopmental disorders

## Abstract

CRISPR/RfxCas13d (CasRx) editing system can specifically and precisely cleave single-strand RNAs, which is a promising treatment for various disorders by downregulation of related gene expression. Here, we tested this RNA-editing approach on Beethoven (*Bth*) mice, an animal model for human DFNA36 due to a point mutation in *Tmc1*. We first screened 30 sgRNAs in cell cultures and found that CasRx with sgRNA3 reduced the *Tmc1*^*Bth*^ transcript by 90.8%, and the *Tmc1* wild type transcript (*Tmc1*^*+*^) by 44.3%. We then injected a newly developed AAV vector (AAV-PHP.eB) based CasRx into the inner ears of neonatal *Bth* mice, and we found that *Tmc1*^*Bth*^ was reduced by 70.2% in 2 weeks with few off-target effects in the whole transcriptome. Consistently, we found improved hair cell survival, rescued hair bundle degeneration, and reduced mechanoelectrical transduction current. Importantly, the hearing performance, measured in both ABR and DPOAE thresholds, was improved significantly in all ages over 8 weeks. We, therefore, have validated the CRISPR/CasRx-based RNA editing strategy in treating autosomal-dominant hearing loss, paving way for its further application in many other hereditary diseases in hearing and beyond.

## Introduction

According to the World Health Organization (WHO), hearing loss is one of the most common sensory defects, with approximately 5% of the world’s population suffers from disabling hearing loss, and 34 million of these are children.^1,2^ In children, hearing loss affects cognitive, language, and psychosocial development.^1,2^ Almost half of all cases of deafness cases are caused by genetic factors,^3^ and among the different types of hereditary hearing loss, 20–25% of nonsyndromic hearing loss (NSHL) cases are autosomal dominant.^4,5^ To date, over 100 genes have been confirmed to be relevant to NSHL (https://hereditaryhearingloss.org/), and the prevalence of autosomal dominant inheritance increases while that of autosomal recessive inheritance decreases in aging populations.^6^ For example, *TMC1* is the sixth most commonly inherited deafness gene, and mutations in *TMC1* result in both dominant and recessive NSHL.^7^ Its protein product TMC1 is believed to have ten transmembrane domains, and together with TMC2, it forms the pore of a channel complex that is required for mechanoelectrical transduction of sound in both auditory and vestibular hair cells.^8,9^ The *TMC1* point mutation (c.1253T > A; p.M418K) in humans, which is identical to the *Tmc1* mutation (c.1235T > A; p.M412K) in *Bth* mice, a transversion T > A locates in exon 13 of *Tmc1* sequence. This mutation causes DFNA36 hearing loss,^10,11^ so *Bth* mice would be an appropriate model for NSHL research.

Currently, few treatments are available to slow or reverse genetic deafness in clinic.^12^ With an increasing understanding of heredity in relation to hearing loss, interest in gene therapies for hearing loss has grown.^13^ Gene replacement was first used to successfully restore hearing in mice with a null mutation in the gene coding for vesicular glutamate transporter-3 (*VGLUT3*).^14^ Subsequent studies also confirmed the usefulness of gene replacement in treating hereditary hearing loss,^15–19^ but gene replacement cannot precisely regulate gene expression according to the needs of the cells and it would be less effective when the mutant transcript is dominant-negative. Gene editing technology as a novel method of gene therapy has been applied to the treatment of genetic hearing loss, and delivering the CRISPR/Cas system into the inner ear successfully ameliorates hearing loss in *Bth* model mice.^20–22^ In addition, packaging cytosine base editors into dual AAVs restored gene function in Baringo mice that carry a recessive loss-of-function point mutation in the *Tmc1* gene, and this demonstrated that in vivo base editing could partially and transiently rescue auditory function.^23,24^ However, genome editing might induce off-target mutations in DNA sequences that are similar to the targeting sequence, which limits the usefulness of such technology, especially for therapeutic and clinical applications.^25,26^ Genetic therapies on the RNA level, however, only modify the expression of target RNA without affecting the DNA. In recent years, RNA regulation has been used to treat hearing loss in mice. For example, antisense oligonucleotides were applied in a mouse model to rescue the inner ear mutation of *Ush1c* (c.216G > A), the splice variant causes disruption of wild-type splicing, which results in a frameshift and a truncated protein;^27^ RNA interference and artificial microRNA reduced RNA expression and protected against hearing loss.^28–30^ However, widespread off-target transcript silencing by these traditional RNA regulating tools has been a consistent concern.^31,32^

CRISPR/Cas13, as a new RNA interference tool, is a class 2 type VI CRISPR/Cas RNA endonuclease initially used to mitigate viral infection in bacteria,^33^ and it has higher specificity than traditional RNA interference tools.^34^ Four members of the Cas13 protein family have been identified, including Cas13a (previously known as C2c2),^35^ Cas13b,^36^ Cas13c,^37^, and Cas13d.^38^ It has been reported that PspCas13b and CasRx have higher activity and specificity than other Cas13s.^36,38^ As the most compact Cas13 enzymes at present, CasRx can be easily packaged into AAVs,^12^ and this makes it convenient to deliver the CRISPR/CasRx system in vivo. CasRx has been applied as a therapeutic tool in mouse models of liver and eye ailments,^39–41^ and compared to other gene editing systems the RNA editing system can provide a much safer approach for gene silencing without permanently altering the genome.^42^ In addition, the protospacer flanking sequence (PFS) is necessary for most Cas13s, limiting the selection of sgRNA sequences, especially for specific pathogenic point mutations. In contrast, there is no PFS restriction in the CasRx system, and thus a broader array of gRNAs can be designed and screened.

There is still a lack of studies using the CRISPR/Cas13 RNA editing system for hereditary deafness therapy. To explore the potential therapeutic effects of CRISPR/Cas13, we screened 30 sgRNAs that match the single point mutation at all possible positions to target the pathogenic allele of *Tmc1*, and we compared the editing specificity and efficiency between PspCas13b and the CasRx system to select an optimal sgRNA. We used a newly developed AAV vector, AAV-PHP.eB,^43^ with high transduction efficiency for inner ear hair cells to deliver CasRx and sgRNA to the natal mouse cochlea and successfully downregulated the expression of the *Tmc1*^*Bth*^ transcript. The change of mRNA expression ratio of *Tmc1*^*Bth*^/*Tmc1*^*+*^, prevented progressive hearing loss, and improved the morphology of hair cells and stereocilia bundles without detectable off-target effects. These results suggest that CasRx RNA editing is a potential clinical approach for treating genetic deafness.

## Results

### Specific knockdown of the *Tmc1*^*Bth*^ transcript in vitro using CasRx

**Fig. 1:**
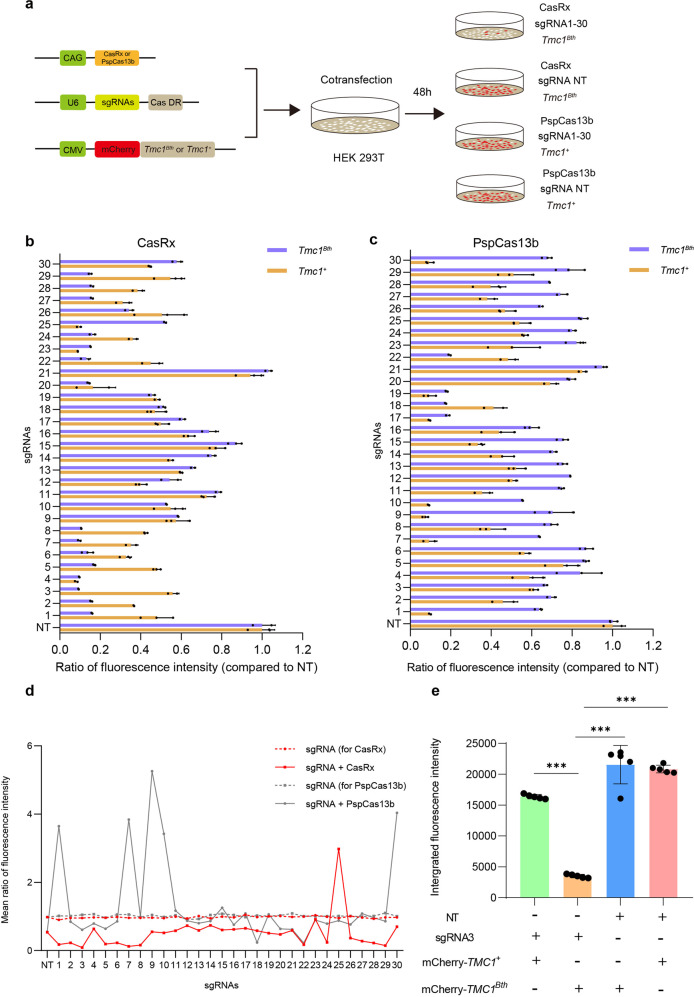
Screening for efficient and specific sgRNAs for targeting the *Tmc1*^*Bth*^ transcript. **a** Constructs used for the sgRNA screen mediated by the Cas RNA editing system. Five vectors were constructed, including the Cas expression vector, the mCherry-*Tmc1*^*Bth*^ fluorescence reporter, the mCherry-*Tmc1*^*+*^ fluorescence reporter, the sgRNA expression vectors for targeting the *Tmc1*^*Bth*^ transcript, and the non-targeting (NT) sgRNA expression vector. **b** Ratios of fluorescence intensity with sgRNAs compared to control sgRNA (NT) for targeting mCherry-*Tmc1*^*Bth*^ mRNA and mCherry-*Tmc1*^*+*^ mRNA mediated by CasRx system. Data are shown as the mean ± SD (*n* = 3 biologically independent samples). **c** Ratios of fluorescence intensity with sgRNAs compared to control sgRNA (NT) for targeting mCherry-*Tmc1*^*Bth*^ mRNA and mCherry-*Tmc1*^*+*^ mRNA mediated by PspCas13b system. Data are shown as the mean ± SD (*n* = 3 biologically independent samples). **d** Mean ratio of fluorescence intensities between mCherry-*Tmc1*^*Bth*^ and mCherry-*Tmc1*^*+*^ mRNA. sgRNA3 has the lowest mean ratio of fluorescence intensity for all the 30 sgRNAs tested. **e** The integrated fluorescence intensity of cells with CasRx system. The integrated fluorescence density was significantly decreased with sgRNA3 targeting mCherry-*Tmc1*^*Bth*^ mRNA compared to targeting mCherry-*Tmc1*^*+*^ mRNA, and integrated fluorescence density was decreased comparted to targeting mCherry-*Tmc1*^*Bth*^ and mCherry-*Tmc1*^*+*^ mRNA with non-targeting sgRNA. Data are shown as the mean ± SD (*n* = 5 biologically independent samples). ***p < 0.001, *P*-values were determined by one-way ANOVA with Sidak’s multiple comparisons test

We aimed to disrupt *Tmc1*^*Bth*^ *m*RNA with high efficiency and specificity in HEK 293T cells. We compared two RNA editing systems, PspCas13b and CasRx, both of which have been shown to efficiently knock-down endogenous transcripts.^36,38^ We designed 30 sgRNAs targeting *Tmc1*^*Bth*^ RNA because the lengths of the sgRNAs were 30 bp in both the PspCas13b and CasRx systems, and a 30 bp sgRNA (NT) targeting EGFP was used as control. The point mutation A was presented in first to 30th of the 30 bp sequence. (Supplementary Fig. S1 and Supplementary Table S1). To screen for the optimal sgRNA, we constructed two mCherry fluorescence reporters that contained wild type or mutant *Tmc1* sequence with the mCherry gene fused at the 5′ end of the sequence (Fig. 1a). We co-transfected the reporters, the PspCas13b or CasRx expression vectors, and the sgRNA expression vectors into 293T cells, and after 48 h of transfection the RNA editing system disrupted the expression of the fused RNA and the fluorescence intensity of the cells was measured as an indication of the RNA knockdown efficiency. Next, the ratio of the averaged fluorescence intensities was calculated between targeted and non-targeted sgRNA cell wells (Fig. 1b, c), for mCherry-*Tmc1*^*Bth*^ or mCherry-*Tmc1*^*+*^ cell wells. Lower ratio indicates higher targeting specificity. As expected, CasRx and PspCas13b resulted in a dramatic decrease in mCherry expression in 293T cells (Supplementary Fig. S2). The lowest ratios of fluorescence intensities of the cells were 9.2 ± 0.13% and 17.44 ± 0.48% when using the CasRx and PspCas13b systems, respectively, to target mCherry-*Tmc1*^*Bth*^ (Fig. 1b, c). Further, in order to compare the specificity of the two systems, we measured the mean fluorescence intensities by targeting mCherry-*Tmc1*^*+*^, and we analyzed the ratio of the mean fluorescence intensities between mCherry-*Tmc1*^*Bth*^ and mCherry-*Tmc1*^*+*^ mRNA interference (Fig. 1d). The sgRNA3 in the CasRx system exhibited the lowest ratio at 0.089113, which decreased about 90.8% mCherry-*Tmc1*^*Bth*^ and 44.3% mCherry-*Tmc1*^*+*^ mRNA respectively (Fig. 1d). The additional experiment results showed in Fig. S3, the knockdown efficiency of sgRNA3-mediated knockdown of *Tmc1*^*Bth*^ and *Tmc1*^+^ were 91.9% and 43.4%, respectively (Supplementary Fig. S3a), and the ratio was 0.07854677 (Supplementary Fig. S3b). The results showed that mCherry integrated density significantly decreased by 83.7% which was mediated by sgRNA3 in CasRx system (Fig. 1e), indicating the efficient knock-down of the *Tmc1*^*Bth*^ transcript. Taken together, these results demonstrated that sgRNA3 in CasRx was the ideal sgRNA for efficient knockdown of the *Tmc1*^*Bth*^ transcript with significantly less knockdown of *Tmc1*^*+*^ transcript.

### Off-target analysis of CasRx-mediated RNA knockdown in 293T cells

**Fig. 2:**
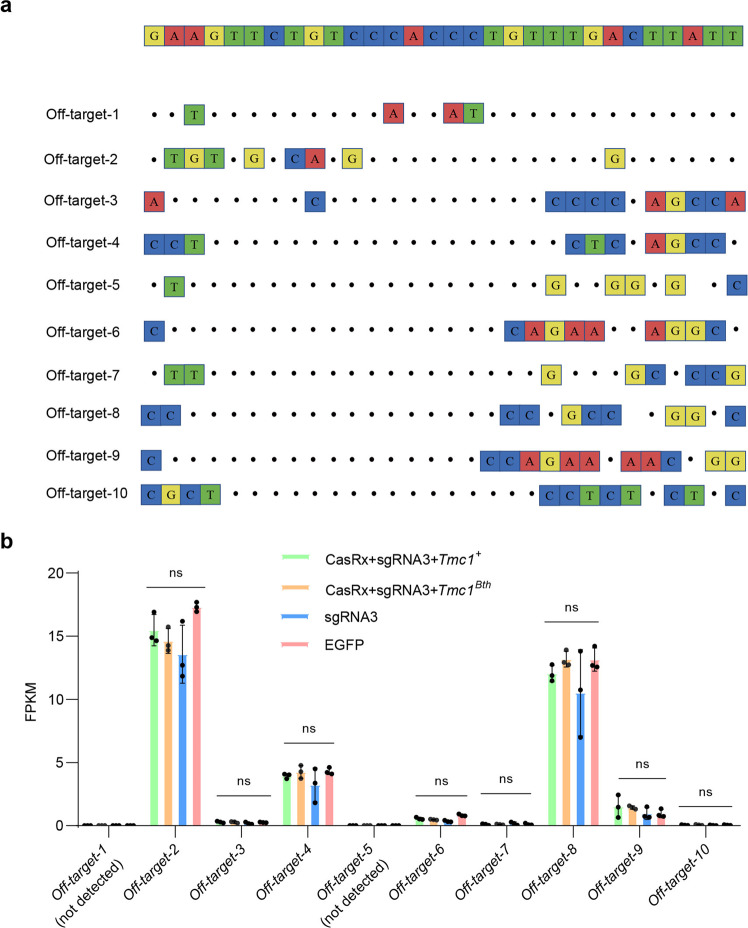
Off-target analysis for RNA editing in 293T cells by RNA-Seq. **a** Off-target-1 to Off-target-10 are ten off-target sites detected by RNA-seq. Mismatches compared to the on-target site are shown and highlighted in color. The 30 bp sequence (On-target) targeted by the sgRNA3 is shown in the top row. **b** Off-target analysis for RNA editing in 293T cells by RNA-Seq. No significant difference was found in CasRx + sgRNA3 + mCherry-*Tmc1*^*Bth*^, CasRx + sgRNA3 + mCherry-*Tmc1*^*+*^ or sgRNA3, when compared to EGFP. Data are shown as the mean ± SD. ns no significance. Statistical analysis was performed by multiple unpaired t-test

Four experiment groups were conducted, including CasRx + sgRNA3 + mCherry-*Tmc1*^*Bth*^, CasRx + sgRNA3 + mCherry-*Tmc1*^*+*^, sgRNA3 and EGFP, 3 replicates per group. 5 × 10^6^ EGFP^+^ positive cells per sample were collected by FACS. Then we extracted the total RNA of these cells for RNA-Sequencing. We first examined the RNA-Sequencing data at the whole genome level, and we found the gene expression profiles of both CasRx + sgRNA3 + mCherry-*Tmc1*^*Bth*^ and CasRx + sgRNA3 + mCherry-*Tmc1*^*+*^ are comparable to that of EGFP (Supplementary Fig. S4). To further quantify possible off-target effects, we screened the top 10 most likely off-target genes according to the 30 bp sgRNA sequence by aligning on human whole genome (Fig. 2a). Among these 10 genes, two were not detectable in expression for all groups. For the other 8 genes, we found no significant expression difference in CasRx + sgRNA3 + mCherry-*Tmc1*^*Bth*^, CasRx + sgRNA3 + mCherry-*Tmc1*^*+*^ or sgRNA3, when compared to EGFP (Fig. 2b and Supplementary Table S4). These results suggested that CasRx-mediated RNA knockdown had no off-targets in 293T cells.

### Specific targeting mediated by CasRx in vivo

**Fig. 3:**
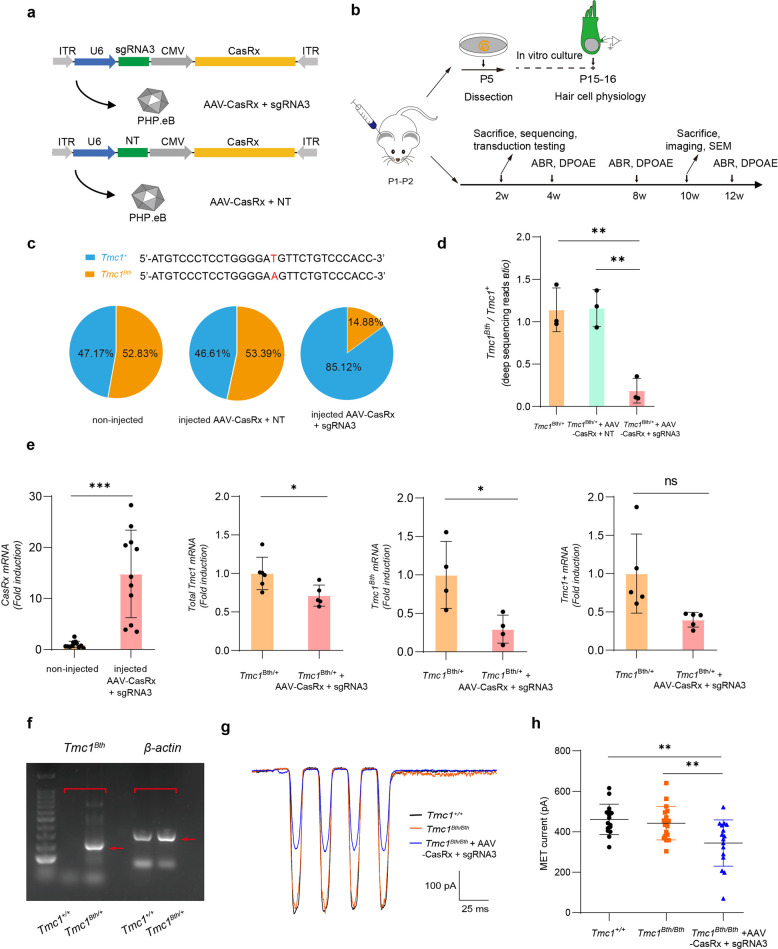
CasRx selectively disrupts the *Tmc1*^*Bth*^ transcript in *Bth* mice. **a** Schematic of the AAV vector encoding CasRx and sgRNA3 (upper), and a control NT vector (lower). **b** Outline of the in vivo experiments. Mice were injected with AAV (~5 × 10^9^ vg) at P1–P2, and the organs of Corti were dissected and cultured at P5, and hair cell physiology was analyzed at P15–P16. Injected mice were sequenced after 2 weeks followed by hearing tests (ABR and DPOAE) after 4, 8, and 12 weeks, immunohistochemistry, and scanning electron microscopy at 10 weeks after injection. **c** The percentage of deep sequencing reads of *Tmc1*^*Bth*^ and *Tmc1*^*+*^. Pie charts indicate the mean composition of *Tmc1*^*Bth*^ and *Tmc1*^*+*^ transcripts in these samples, sequences show the single-nucleotide difference between the *Tmc1*^*Bth*^ and *Tmc1*^*+*^ transcripts (52.83 ± 5.33%, 53.39 ± 4.8%, and 14.88 ± 9.77% *Tmc1*^*Bth*^ transcript for non-injected, injected with AAV-CasRx + NT, and injected with AAV-CasRx + sgRNA3, respectively. *n* = 3 mice, data are shown as the mean ± SD). **d** Deep sequencing analysis of the ratios of transcripts between *Tmc1*^*Bth*^ and *Tmc1*^*+*^ for non-injected mice (*n* = 3 mice), mice injected with AAV-CasRx + NT (*n* = 3 mice), and mice injected with AAV-CasRx + sgRNA3 (*n* = 3 mice), respectively. Data are shown as the mean ± SD, **p < 0.01, *P*-values were determined by one-way ANOVA with Dunnett’s multiple comparisons test. **e** mRNA expressions in the cochlea at 2 weeks after injection as measured by RT-qPCR. The expression of CasRx mRNA (*n* = 11 mice), total *Tmc1* mRNA (*n* = 5 mice), *Tmc1*^*Bth*^ mRNA (*n* = 4 mice), and *Tmc1*^*Bth*^ (*n* = 5 mice) between injected with AAV-CasRx + sgRNA3 and non-injected contralateral ears were showed in graphs. Relative mRNA expression levels were calculated with the ΔΔCt algorithm. Data are shown as the mean ± SD, *p < 0.05, ***p < 0.001, *P*-value was determined by unpaired two-tailed *t*-test. **f** Amplification of the *Tmc1*^*Bth*^ sequence. The amplicon was detected by a pair of specific targeting primers with heterozygous templates, and the primers cannot amplify with the wild-type template. **g** Representative MET recordings and maximal MET current amplitudes of apical IHCs at the equivalent of P15–P16. **h** The MET current amplitude was 461.134 ± 74.978 pA, 442.458 ± 82.805 pA, and 344.409 ± 114.591 pA in *Tmc*^*+/+*^ mice (*n* = 16 OHCs), non-injected *Tmc1*^*Bth/Bth*^ mice (*n* = 18 OHCs), and *Tmc1*^*Bth/Bth*^ mice injected with AAV-CasRx + sgRNA3 (*n* = 16 OHCs), respectively. Data are shown as the mean ± SD, **p < 0.01, *P*-values were determined by one-way ANOVA with Sidak’s multiple comparisons test

Previous results demonstrated that sgRNA3 in the CasRx system had the highest efficiency in targeting *Tmc1*^*Bth*^ transcript in 293T cells, to determine whether AAV-CasRx + sgRNA3 targets *Tmc1*^*Bth*^ in vivo, an AAV vector encoding sgRNA3 and CasRx was used to downregulate the *Tmc1*^*Bth*^ transcript in the inner ears of *Bth* mice, and non-target sgRNA was packaged in the same vector as a control (Fig. 3a). We used the engineered AAV-PHP.eB, which is the more efficient and further evolved AAV variant of the PHP serotype, as the delivery vector.^44^ To validate the ability of AAV-PHP.eB to deliver genes into inner hair cells (IHCs) and outer hair cells (OHCs), we injected AAV-PHP.eB encoding EGFP into the right inner ear of postnatal day (P)1-P2 mice through the round window membrane. The cochleae were collected 2 weeks after injection, and the organs of Corti were dissected for immunohistochemistry (Fig. 3b). We observed a nearly 100% viral transduction efficiency in IHCs and OHCs showed over 95% viral transduction efficiency that decreased from the apical to basal turns (Supplementary Fig. S5a), which was consistent with our previous research.^43^

To determine the editing ability of CasRx in vivo, we performed targeted deep sequencing from whole cochlear tissues. The cochleae from *Bth* mice injected with AAV-CasRx + sgRNA3 showed that 14.88 ± 9.77% of the total *Tmc1* transcripts were *Tmc1*^*Bth*^ transcripts, which was significantly decreased compared to non-injected mice (52.83 ± 5.33%) and mice injected with AAV-casRx + NT (control AAV, non-targeting sgRNA) (53.39 ± 4.8%) (Fig. 3c and Supplementary Table S3). The actual ratios of the *Tmc1*^*Bth*^ and *Tmc1*^*+*^ transcripts were 1.1397 ± 0.2584, 1.1605 ± 0.2183, and 0.186 ± 0.1457 for non-injected, AAV-CasRx + NT, and AAV-CasRx + sgRNA3, respectively (Fig. 3d). This indicated a knockdown efficiency of 70.2% for the *Tmc1*^*Bth*^ transcript.

To expand our analysis of the expression of *Tmc1* in mouse, we used RT-qPCR to measure gene expression at the RNA level. We injected AAV-EGFP and AAV-CasRx + sgRNA3 in wild-type mice, and compared the expression in 2 weeks after injection. Both vectors showed highly expression in injected cochleae comparing to controls (Fig. 3e and Supplementary Fig. S5b). We next injected AAV-CasRx + sgRNA3 in *Bth* mice, and observed decreased expression of total *Tmc1* compared to non-injected contralateral cochleae (Fig. 3e). RNA level of *Tmc1*^*Bth*^ was assessed with a pair of primers to specifically target *Tmc1*^*Bth*^ cDNA (Fig. 3f), and *Tmc1*^*+*^ mRNA level was detected using specific primers by RT-qPCR. Results showed that mRNA levels of *Tmc1*^*+*^ and *Tmc1*^*Bth*^ were decreased to 39.7 and 29.5% of non-injected controls, respectively (Fig. 3e). CasRx editing caused the downregulation of both *Tmc1*^*+*^ and *Tmc1*^*Bth*^ mRNA in injected cochleae, but the mutant transcript decreased to a lower degree.

Next, to confirm the specific targeting of CasRx and sgRNA3 to the mutant *Tmc1* site, we measured the mechanoelectrical transduction (MET) current. The *Tmc1*^*Bth*^ mutation does not affect the sensitivity of hair cell mechanotransduction, but knockdown of *Tmc1* leads to a reduction in MET current.^8,20,21^ *Tmc1* and *Tmc2* are both required for MET,^8,45^ and *Tmc2* is transiently expressed during the first postnatal week and then disappears from the IHC stereocilia at P10, while *Tmc1* is constantly expressed.^45,46^ Therefore, to eliminate the contributions of *Tmc2* to the MET current, IHCs from cochleae at the equivalent of P15-P16 mice were used. We measured the MET current of IHCs from in vitro cultured organs of Corti at the equivalent of P15–P16 (Fig. 3g). The apical IHCs from wild type *Tmc*^*+/+*^ mice and non-injected *Tmc1*^*Bth/Bth*^ mice exhibited similar MET current amplitudes, while the MET current amplitudes were significantly reduced in apical IHCs from *Tmc1*^*Bth/Bth*^ mice injected with AAV-CasRx + sgRNA3 (Fig. 3h). This suggested that CasRx + sgRNA3 could target the *Tmc1*^*Bth*^ mRNA in the mouse inner ear.

Taken together, our results confirm the efficient and selective knockdown of the *Tmc1*^*Bth*^ transcript mediated by AAV-CasRx + sgRNA3 in vivo.

### Prevention of progressive hearing loss by RNA knockdown in vivo

**Fig. 4:**
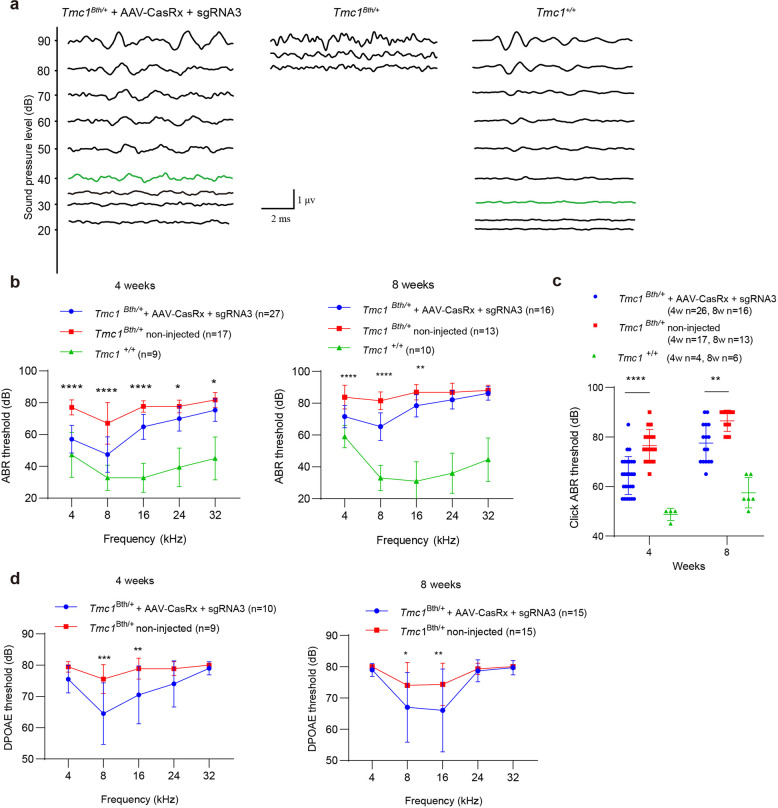
Improvement in auditory function via CasRx in *Bth* mice. **a** ABR waveforms recorded at 8 kHz at 4 weeks in the *Tmc1*^*Bth/+*^ injected right ear, the non-injected left ear, and the wild-type ear. The green traces indicate the threshold. **b**, **c** Tone-burst and click-evoked ABR thresholds at 4, 8 weeks in *Tmc1*^*+/+*^ (Green), *Tmc1*^Bth/+^ + AAV-CasRx + sgRNA3 (Blue), and *Tmc1*^*Bth/+*^ non-injected contralateral (Red) ears. Mean ABR thresholds were significantly reduced in ears injected with AAV-CasRx + sgRNA3 (~5 × 10^9^ vg of AAV) compared to non-injected *Tmc1*^Bth/+^ ears after 4 and 8 weeks. Statistical analysis was performed by two-way ANOVA with Tukey’s post hoc test for multiple comparisons. **d** DPOAE thresholds at 4, 8 weeks in *Tmc1*^*Bth/+*^ + AAV-CasRx + sgRNA3 (Blue) and *Tmc1*^*Bth/+*^ untreated contralateral (Red) ears. DPOAE thresholds were significantly reduced in the ears injected with AAV-CasRx + sgRNA3 (~5 × 10^9^ vg of AAV) at two frequencies. Statistical analysis was performed by two-way Bonferroni’s multiple comparisons test. *p < 0.05, **p < 0.01, ***p < 0.001, and ****p < 0.0001. Values and error bars represent the mean ± SD

The convincing therapeutic effects were obtained because CasRx disrupted the *Tmc1*^*Bth*^ transcript guided by sgRNA3 with less interfering in *Tmc1*^*+*^ transcript. Hearing in *Bth* mice was protected when the *Tmc1*^*Bth*^ transcript was disrupted and the level of the harmful protein decreased, while the control mice injected with AAV encoding a non-targeting (NT) RNA had progressive hearing loss (Supplementary Fig. S6). To measure hearing function in the injected cochleae, we performed auditory brainstem response (ABR) tests every 4 weeks because the *Tmc1* mutation induces progressive hearing loss. We measured tone-burst ABRs at frequencies of 4, 8, 16, 24, and 32 kHz, and ABR waveforms recorded at 8 kHz showed that injection of CasRx + sgRNA3 greatly improved hearing function compared to non-injected controls (Fig. 4a). At the fourth week after injection, *Bth* mice injected with AAV-CasRx + sgRNA3 had lower ABR thresholds at all frequencies (57 ± 9, 47 ± 11, 65 ± 8, 70 ± 8, and 75 ± 7 dB at 4, 8, 16, 24, and 32 kHz, respectively) compared to non-injected contralateral ears (77 ± 5, 67 ± 13, 78 ± 4, 78 ± 4, and 82 ± 5 dB at 4, 8, 16, 24, and 32 kHz, respectively) (Fig. 4b). ABR thresholds of *Bth* mice were not reduced at 4 weeks after injecting AAV-CasRx + NT (Supplementary Fig. S7a), and either AAV-CasRx + sgRNA3 nor AAV-Cas + NT had any impact on hearing in wild type mice (Supplementary Fig. S7b). At 8 weeks after injection, the ABR thresholds rose in both ears, but the treated ear still had lower thresholds at low frequency (72 ± 7, 65 ± 9, and 78 ± 7 dB for 4, 8, and 16 kHz, respectively) compared to non-injected ears (84 ± 7, 82 ± 6, and 87 ± 5 dB for 4, 8, and 16 kHz, respectively) (Fig. 4b).

We further measured click-evoked ABRs and found that the thresholds in the injected ears were significantly decreased at 4 and 8 weeks after injection, which was consistent with the pure tone ABR results showing that AAV-CasRx + sgRNA3 injection slowed the progressive hearing loss in *Bth* mice (Fig. 4c). Click-evoked ABR peak 1 (P1) amplitudes at 80 and 90 dB showed increase in AAV-CasRx + sgRNA3 injected *Bth* mice compared to non-injected mice at 4 weeks, latencies of P1 waves of injected *Bth* mice were also normalized by injection (Supplementary Fig. S7c, d).

We also measured the distortion product otoacoustic emissions (DPOAEs) to evaluate the function of OHCs (Fig. 4d). Ears injected with AAV-CasRx + sgRNA3 showed lower DPOAE thresholds at 8 kHz and 16 kHz at 4 and 8 weeks after injection (65 ± 10 and 71 ± 9 dB for 8 and 16 kHz at 4 weeks and 76 ± 5 and 79 ± 3 dB for 8 and 16 kHz at 8 weeks), while the non-injected ears lacked DPOAE indicating a lack of OHCs function.

These results suggested that mRNA knockdown mediated by AAV-CasRx + sgRNA3 could improve the hearing function over a period 8 weeks.

### Preservation of hair cells and stereocilia bundle morphology mediated by CasRx

**Fig. 5:**
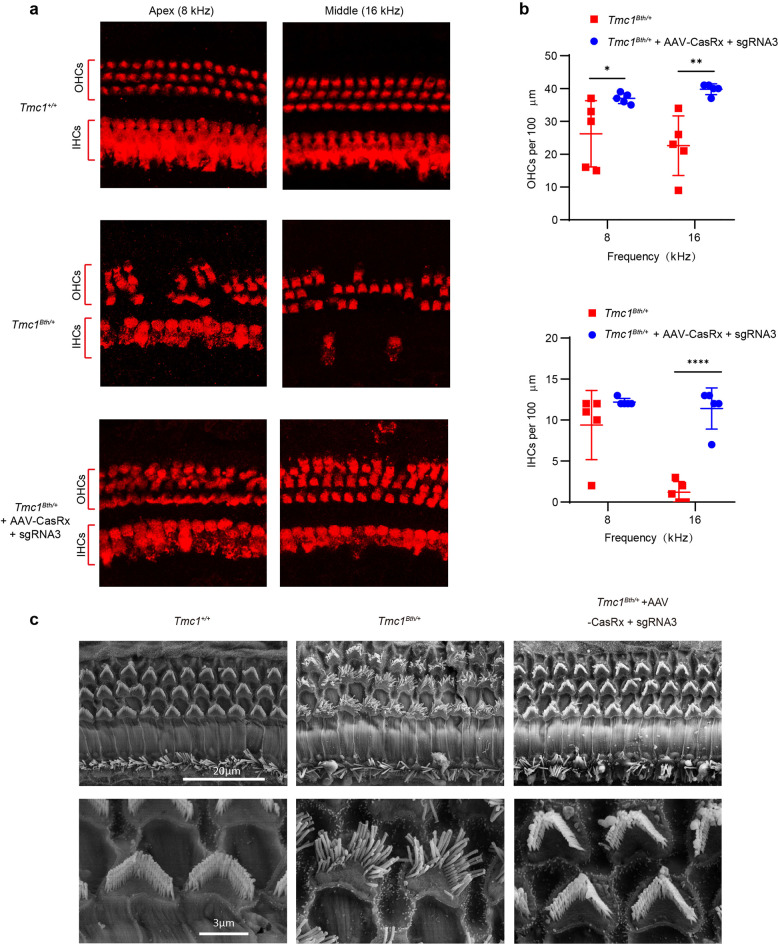
Injection of AAV-CasRx + sgRNA3 (~5 × 10^9^ vg of AAV) protects hair cells and hair bundles. **a** Representative confocal images of 100 μm cochlear sections harvested 10 weeks after injection. Samples were stained with myosin7a (Red). The images are from *Tmc1*^+/+^, *Tmc1*^*Bth/+*^ + AAV-CasRx + sgRNA3, and *Tmc1*^*Bth/+*^ non-injected mice (*n* = 5 mice) at locations corresponding to 8 and 16 kHz. The IHCs and OHCs are indicated. Scale bar: 20 μm. **b** The number of OHCs (upper) and IHCs (lower) per 100 μm of the cochleae. Data are shown as the mean ± SD, *p < 0.05, **p < 0.01, ***p < 0.001, and ****p < 0.0001. Statistical analysis was performed by two-way Sidak’s multiple comparisons test. **c** SEM images of the apical cochlear sensory epithelium showing the morphology of the hair cell bundles. *Tmc1*^+/+^, *Tmc1*^*Bth/+*^ + AAV-CasRx + sgRNA3, and *Tmc1*^*Bth/+*^ non-injected samples were collected 10 weeks after injection. Scale bars: 20 μm (upper); 3 μm (lower)

To determine whether CasRx and sgRNA3 can preserve hair cells and hair bundle morphology, we sacrificed the mice at 10 weeks of age and performed confocal and scanning electron microscopy (SEM) analyses. We found that OHCs in the apical turn of the organ of Corti (8 kHz region) began to be lost, and OHC loss became more severe from the middle turn (16 kHz region) to the basal turn (32 kHz region), where the OHCs were almost completely absent (Fig. 5a). IHCs in the apical turn remained intact, while some IHCs were lost in the middle turn and IHCs were completely absent in the basal turn (Fig. 5a). These results were consistent with those of previous studies.^10,20^ In the cochleae injected with AAV-CasRx + sgRNA3, the survival of both IHCs and OHCs was improved, and the number of OHCs per 100 μm of the cochlea increased in the 8 and 16 kHz regions (37.0 ± 1.6, 39.8 ± 1.6 compared to 26.2 ± 10.1, 22.6 ± 9.1) while the IHC number increased in the 16 kHz region (11.4 ± 2.5 compared to 1.2 ± 1.3) (Fig. 5b).

We next used SEM to evaluate the hair bundle morphology. The OHCs and IHCs of wild-type *Tmc1*^*+/+*^ mice at 10 weeks of age showed neatly arranged hair bundles, while the non-injected mice showed severe bundle disorganization. Hair cells injected with AAV-CasRx + sgRNA3 had normal IHC and OHC morphology in the apical turn (Fig. 5c), and hair bundles were preserved in the middle region (Supplementary Fig. S8). These results were consistent with the ABR data showing the protection of hearing in the lower frequency region (4–8 kHz).

### Off-target analysis of CasRx-mediated RNA knockdown in vivo

**Fig. 6:**
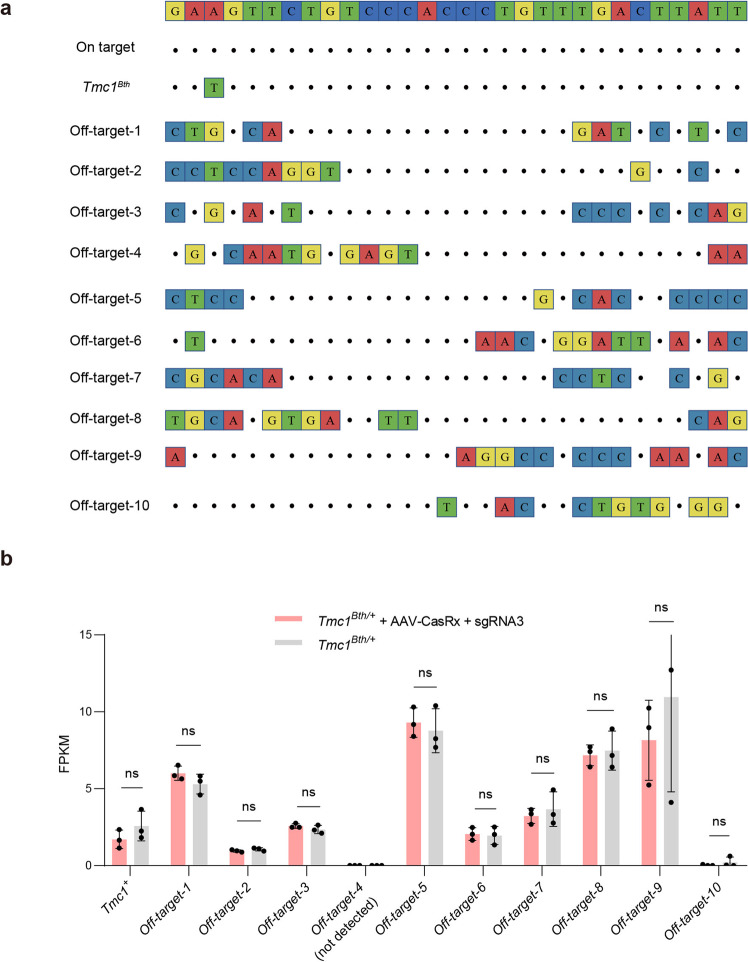
Off-target analysis for RNA editing in vivo by RNA-Seq. **a** Off-target-1 to Off-target-10 were ten off-target sites detected by RNA-seq. Mismatches compared to the on-target site are shown and highlighted in color. The 30 bp sequence (On-target) targeted by the sgRNA3 is shown in the top row. **b** Comparison of FPKM values of the ten off-target sites at injected AAV-CasRx + sgRNA3 or non-injected ears. Data are shown as the mean ± SD. ns no significance. Statistical analysis was performed by multiple unpaired *t*-test

We performed RNA-seq in the cochleae collected 2 weeks after AAV injection. We screened the top 10 most likely off-target genes according to the 30 bp sgRNA sequence by aligning on mouse whole genome (Fig. 6a), and we analyzed the expression difference between the AAV-CasRx + sgRNA3-injected (*n* = 3 mice) and non-injected (*n* = 3 mice) groups. There was no difference in RNA expression for 9 of the 10 genes, one gene (*Gm13492*) was not detected (Fig. 6b and Supplementary Table S4). Nonetheless, the RNA expression differences were limited, suggesting that CasRx-mediated RNA knockdown had few off-target effects.

## Discussion

*TMC1* mutations account for 4–8% of all cases of heritable hearing loss in the world.^7,47,48^ In this study, we used the CRISPR/CasRx system to downregulate the *Tmc1*^*Bth*^ mRNA transcript in the *Bth* mouse model of human genetic deafness with less knockdown of *Tmc1*^*+*^ transcript (there is only a single nucleobase difference between the two transcripts) both in vitro and in vivo. The new, highly efficient AAV-PHP.eB delivery system targeting for hair cells packaged CasRx and sgRNA3 was delivered into the cochlea of *Bth* mice, resulting in preservation of auditory function due to the preservation of hair cell survival and rescue of hair bundle degeneration. These results demonstrate that the CasRx system can successfully ameliorate dominant-negative hearing loss by specifically knocking down the mutant transcript.

The CasRx RNA editing system showed high knockdown efficiency in our study, and co-transfection of vectors encoding the exogenous *Tmc1* sequence, CasRx, and sgRNA into 293T cells resulted in more than 80% knockdown of the *Tmc1*^*Bth*^ mRNA transcripts. CasRx RNA knockdown also showed over 70% RNA knockdown in vivo, and two main factors contributed to the high efficiency. First, CasRx is the smallest protein among the Cas13 family of proteins, which makes it possible to package into a single AAV. Second, different AAV serotypes induce different transduction efficiencies, and several evolved AAVs have been confirmed to be safe and efficient for delivering genes into the inner ear.^49,50^ We previously demonstrated that the AAV-PHP.eB vector had extremely high transduction efficiency in cochlear IHCs and OHCs in vivo.^43^ The vector has potential to deliver CasRx and sgRNA to hair cells with a transduction efficiency of over 95% (Supplementary Fig. S5a). Previous study showed Cas9 and sgRNA induced target gene disruption at about 25% efficiency in hair cells following RNP delivery, indicating that AAV-PHP.eB is more effective compared to non-viral vector.^20^

CasRx-mediated RNA knockdown ameliorated the auditory function in *Bth* mice. In our results, we detected the hearing was improved about 10–20 dB at 4 and 8 weeks in *Bth* mouse, the hearing loss was still be prevented in 12 weeks at low frequencies (data not shown), our data were consistent and comparable with the amelioration effects by SaCas9 based DNA editing technology which showed an improved ABR function over 12 weeks after injection.^21^ The numbers of IHCs and OHCs in the treated ear were greater than in the untreated ears, which was generally consistent with the improved hearing. In the apical turn, the stereocilia bundles were disorganized in the *Bth* mouse model, and this prevented sound from being transmitted thus resulting in higher ABR thresholds at low frequency. This explains why the hearing thresholds at low frequencies were high even though the hair cells were present. We demonstrated that the hair bundle morphology of IHCs and OHCs in injected ears was much better than non-injected ears in apical turn and the hearing was preserved very well at low frequency. Furthermore, injection with control AAV (AAV-CasRx + NT) didn’t rescue hearing on the heterogeneous mouse ears (Supplementary Fig. S7a), then we injected AAV-CasRx + sgRNA3 and control AAV on wild-type mouse and found that no ABR threshold shifts were observed comparing with non-injected ears (Supplementary Fig. S7b), indicating that the hearing loss was prevented by AAV-CasRx + sgRNA3 editing specifically, the injection of AAV-CasRx + sgRNA3 was safe and it did not affect normal hearing function. Despite our positive results, we found that auditory function was still not completely rescued by injecting AAV-CasRx + sgRNA3 compared to wild-type mice especially in the high-frequency region, explanations for the results include that degeneration of hair cells begins in the base and spreads apically, and the efficiency of RNA knockdown is relative lower to overcome the dominant-negative effects of mutant *Tmc1* protein in the basal turn compared to apical turn. Although the knockdown efficiency of mutant transcript is high, the interfering of wild-type transcript can not be ignored, and RNA base editing technology with improved precision and specificity remains to developed to directly convert the mutant base into normal, hopefully in the near future. Since the space of cochlea is limited, introducing more volume is impossible, a research team developed a surgical approach of injection route by round window membrane injection combining with semi-circular canal fenestration.^28^ We might need to improve the targeting efficiency for better recovery of auditory function by multiple-site injection in cochlea and selecting more effective AAV vector to improve the transduction efficiency in the basal turn. Another question needs to be explored more systematically is the window of injection time for effective rescuring of hearing function, as this type of dominant-negative hearing loss is postlingual and progressive, leaving a large window for therapy. The neonatal mouse inner ear is partly developed and is equivalent to human cochlea at 26-week gestational age,^51^ so that injection in mouse inner ear in a later postnatal time is necessary to investigate the protective effects in clinical practice. The previous report showed hearing function was improved by injection AAV-mediatd RNAi in *Bth* mouse at ages before 8 weeks, but protective effects degraded when treatment-time was late.^28^ Whether injection of CasRx in juvenile and adult mice could prevent hearing loss is needed for future work.

RNA editing based on the CasRx system has some advantages in disease treatment. At the RNA expression level, CasRx-mediated knockdown has been shown to substantially reduce off-target effects compared to RNA interference knockdown,^31,34,36,38^ and we didn’t detect any off-target effects in vitro and in vivo. At the gene editing level, using the CRISPR system to target RNA can avoid the risks associated with permanent DNA alternation.^42,52^ In our study, we compared the CasRx and PspCas13b systems and found that CasRx had higher efficiency (Fig. 1b, c) and specificity (Fig. 1d). The potential toxic effects of CasRx system in vivo are difficult to be ruled out completely, but we did not detect anything significant. Consistently, a recent study showed that CasRx displayed no toxic effects by injection with up to 300 pg mRNA encoding CasRx protein into per zebrafish embryo, and in mouse embryos, injection with up to 25–50 pg CasRx together with sgRNA to downregulate target mRNA is tolerated without deleterious effects.^53^ The potential toxic effects of CasRx system delivered by AAV was also evaluated in vivo, study tested the knockdown effect via tail-vein injection of CasRx and sgRNA packaged in AAV8, the target mRNA level was significantly reduced after AAV infection in mouse liver, but the level of serum ALT and AST was similar in the AAV-injected mice and non-injected mice, indicating that CasRx system delivered by AAV had no toxic effects in vivo.^37^ These findings suggest that the CasRx system might be a safe and highly efficient RNA knockdown tool when applied in clinical therapeutics in the future.

In conclusion, 85% of all human autosomal-dominant non-syndromic hearing loss is caused by missense mutation,^54^ RNA knockdown strategy could be broadly applicable to this type of hearing loss. we applied CasRx RNA knockdown to prevent hearing loss in the *Bth* mouse model as a proof-of-concept study. It may also be worth determining whether the CasRx system is effective at treating deafness in adult mice. Anyway, we conclude that CasRx has great potential in treating human dominant-negative hearing loss in the future.

## Materials and methods

### Plasmids

The human codon-optimized CasRx gene was synthesized and cloned into a mammalian expression vector with two NLS (Nuclear localization sequence) under the control of the CAG promoter, and the human codon-optimized PspCas13b gene was synthesized and cloned into a mammalian expression vector with a NES (Nuclear export sequence) under control of the CAG promoter.

We then constructed the CasRx sgRNA cloning backbone, which contains two direct repeats for cloning with BspQI enzyme, and constructed the PspCas13b sgRNA cloning backbone, which contains a 3′ direct repeat for cloning with BbsI enzyme. We synthesized sgRNAs as single-stranded DNA oligos. The sgRNA oligos were annealed and cloned under the U6 promoter using the BspQI enzyme in an sgRNA expression vector containing the U6 promoter element and two BspQI enzyme sites for the CasRx system, and they were cloned under the U6 promoter using the BbsI enzyme in an sgRNA expression vector containing the U6 promoter element and two BbsI enzyme sites for the PspCas13b system.

To construct the mCherry-*Tmc1*^*Bth*^ reporter vector, 90 bp sequences of the *Tmc1* transcript that contained the c.1235T > A mutation were synthesized and cloned into the 3′ end of the mCherry gene in which the stop codon had been removed. To construct the mCherry-*Tmc1*^*+*^ reporter vector, 90 bp sequences of the *Tmc1* transcript that contained c.1235T were synthesized and cloned into the 3′ end of the mCherry gene in which the stop codon had been removed (Supplementary Table 2).

### Cells culture and transfection

We cultured 293T cells in Dulbecco’s Modified Eagle Medium (Gibco) supplemented with 10% fetal bovine serum (FBS) (v/v) (Gemini) at 37 °C with 5% CO_2_ under humidified conditions. Before transfection, cells were seeded on poly-D-lysine-coated 24-well plates and maintained at approximately 60–70% confluence. Cells were transfected using EZ Trans Reagent (Shanghai Life iLab) according to the manufacturer’s protocols. For transfection, CasRx or PspCas13b-expressing plasmid (600 ng), sgRNA-expressing plasmid (300 ng), and mCherry-*Tmc1*^*Bth*^ or mCherry-*Tmc1*^*+*^ reporter were mixed in each well. A total of 1 µg DNA and 3 µL EZ Trans Reagent were diluted in 40 µL DMEM separately. The diluted EZ Trans Reagent was then added to the diluted DNA solution, mixed gently, and incubated for 15 min at room temperature to form DNA-EZ Trans Reagent complexes. The DNA-EZ Trans Reagent complexes were then directly added to each well and mixed gently by rocking the plate back and forth. At 6 h post-transfection, the complexes were removed and 0.5 mL complete growth medium was added to the cells.

### Fluorescence-activated cell sorting (FACS)

293T cells were harvested and subjected to flow cytometry 48 h after transfection. mCherry signals were immediately detected on a BD LSRFortessa flow cytometer (BD Biosciences) with FCS Express 5 software (De Novo Software). A total of 10,000 cell events were collected and analyzed with the FlowJo software for each sample.

### Integrated fluorescence intensity

All fluorescence images were acquired with a Nikon Ti-E microscope (Tokyo, Japan), and the Image J software (National Institutes of Health, Bethesda, Maryland, USA) was used to analyze the integrated fluorescence densities of mCherry. At 48 h post-transfection, we captured images of three randomly selected 1300 × 1300 μm regions in each cell well. We then used Image J to extract fluorescence intensity value from all cells in the region, which was averaged across three regions for each cell well. Use image J software to quantitatively analyze fluorescence intensity steps: Firstly, overlay all the 2D diagrams and select max intensity. Secondly, adjust threshold selects all the area of visual field of the cell image for calculation, and the selected area is highlighted in red. Subtract the background until the red highlighted plaque is the desired cell. Finally, calculated the mean integrated fluorescence intensity of each well cells and statistics the ratio between targeted and non-targeted sgRNA cell wells, for mCherry-*Tmc1*^*Bth*^ or mCherry-*Tmc1*^*+*^ cell wells.

### Virus production

AAV (PHP.eB serotype) viral vectors were produced by OBIO Technology Corp., Ltd. (Shanghai, China). AAV carrying a dual transgene cassette, a U6 promoter-driven sgRNA targeting *Tmc1*, and a CMV-driven RfxCas13 promoter were packaged as therapeutic vectors. The control AAV was constructed consistently with the therapeutic vector except that the sgRNA sequence was replaced by a NT sequence. The same serotype AAV encoding an EGFP tag was used for testing the transduction efficiency. Viral titers were 3.38 × 10^12^ vg/ml for AAV-CasRx + sgRNA3 and 1.73 × 10^12^ vg/mL for AAV-CasRx + NT. Virus aliquots were separated into small volumes and stored at –80 °C to avoid repeated freeze-thaw cycles.

### Mice

All animals were bred and housed in our facilities with a 12 h light-dark cycle. Heterozygous Beethoven mice (*Tmc1*^Bth/+^) were obtained as a gift from Dr. Andrew Griffith (Department of Human Genetics and Molecular Medicine, Sackler School of Medicine, Tel Aviv University). *Tmc1*^Bth/+^ mice were inbred with *Tmc1*^*+/+*^ or *Tmc1*^Bth/+^ C3HeB/FeJ (C3H) background mice (Jackson Laboratories) to propagate new pups. DNA was extracted from tail-clip biopsies using lysis solution mixed with proteinase-K at 55 °C for 8 h and then at 85 °C for 1 h, and genotyping was performed by PCR with primers in a 20 μL volume according to a previously published protocol^30^.

### Inner ear injection

*Tmc1*^Bth/+^ or wild-type mouse pups were injected between P1 and P2 with 1.5 μL virus (~5 × 10^9^ vg) via the round window membrane. Pups were anesthetized on ice for 2–3 min until loss of consciousness. Upon anesthesia, a post-auricular incision was made to expose the otic bulla and visualize the round window membrane. Virus was slowly microinjected into the right cochlea using a glass micropipette. After injection, the skin incision was closed using a suture, and the pup was placed on a 42 °C heating pad for recovery. Pups were returned to the parental cage after they fully recovered.

### Hair cell electrophysiology

Cochleae of *Tmc1*^*+/+*^, *Tmc1*^*Bth/Bth*^, or *Tmc1*^*Bth/Bth*^ injected mice were harvested at P4-5 and cultured in DMEM (1X) medium with 1% FBS at 37 °C in a 5% CO_2_ atmosphere. Whole-cell patch-clamp recording was performed in IHCs at P14–15, in a standard artificial perilymph solution containing (in mM) 137 NaCl, 5.8 KCl, 1.3 CaCl_2_, 0.9 MgCl_2_, 0.7 NaH_2_PO_4_, 10 HEPES, and 5.6 D-glucose, with pH adjusted to 7.40 and osmolarity to ~300 mmol/kg. Recording pipettes were pulled from borosilicate glass capillaries (1B150F-4, World Precision Instruments Inc., Florida, USA) and filled with an internal solution containing (in mM) 140 CsCl, 0.1 EGTA, 1 MgCl_2_, 10 HEPES, 2 Mg-ATP and 0.3 Na-ATP (pH 7.20, ~295 mmol/kg). Mechanoelectrical transduction (MET) currents were recorded under voltage-clamp with a holding potential of −80 mV, through an EPC10/2 amplifier (HEKA, Lambrecht/Pfalz, Germany) driven by a PC running Patchmaster (HEKA). Current signals were filtered at 2 kHz and digitized at 200 kHz. Hair bundles were deflected with a fluid jet, delivered through a pipette with a tip of ~10 μm, positioned ~15 μm away.^55^ The fluid jet was driven by a piezoelectric disc (27 mm in diameter) with sinusoidal voltage commands applied (40 Hz, ±120 V).

### Targeted deep-sequencing data analysis

To analyze the CasRx knockdown mutant sequence at the RNA level, total RNA from the cochleae and cDNA were obtained as described above. Target site sequences were amplified with primers TMC1-lib-F and TMC1-lib-R (Supplementary Table 2). PCR products were visualized on 2% agarose gels and purified with a purification kit (Qiagen). Paired-end reads (150 base pairs) were generated on an Illumina MiSeq platform. The reads from heterozygous samples were segregated based on the presence of the wild-type sequence (5′-ATG CCT CCT GGG GAT GTT CTG TCC CAC C-3′ and its reverse complement 5′-GGT GGG ACA GAA CAT CCC CAG GAG GGA CAT-3′) and the mutant sequence (5′-ATG TCC CTC CTG GGG AAG TTC TGT CCC ACC-3′) and its reverse complement 5′-GGT GGG ACA GAA CTT CCC CAG GAG GGA CAT-3′). The knockdown efficiency was calculated as (heoretical proportion – actual proportion)/theoretical proportion) × 100%.

### RT-qPCR of the cochlea

Cochleae from wild-type or heterozygous mice were dissected. Total mRNA was extracted from the organs of Corti by TRIzol (Invitrogen) and mRNA was reverse-transcribed using a cDNA synthesis supermix (YEASEN) according to the manufacturer’s protocol. One microliter of RT product was added to RT-qPCR SYBR (YEASEN) for subsequent RT-qPCR with the following steps: 95 °C for 5 min and 40 cycles of 95 °C for 10 s and 60 °C for 35 s. Primers were designed to detect the total *Tmc1* expression level: q-TMC1-F2 and q-TMC1-R2. To detect the mutant transcript expression, the forward primer, q-TMC1-F4 was designed with a 3′ end ‘A’ that specifically bound to the mutant sequence; to detect the wild-type transcript expression, the forward primer, q-TMC1-F5 was designed with a 3′ end ‘T’ that specifically bound to the wild-type sequence, and q-TMC1-R2 was used as reverse primer.

### Off-target analysis

For analyzing off-target RNA editing sites across the transcriptome, total RNA from different treatment samples was harvested using the RNeasy Plus Miniprep kit (Qiagen). A total of 1 µg RNA per sample was used as the input material for the RNA sample preparations. Sequencing libraries were generated using RNA Library Prep Kit for Illumina^®^ (NEB, USA) following the manufacturer’s recommendations, and index codes were added to attribute sequences to each sample. There were at least 5 million reads per sample. Differential expression analysis of two groups was performed using the DESeq2 R package. DESeq2 provides statistical routines for determining differential expression in digital gene expression data using a model based on the negative binomial distribution. The resulting *P*-values were adjusted using the Benjamini and Hochberg approach for controlling the false discovery rate. Genes with an adjusted *P*-value <0.05 found by DESeq2 were considered to be differentially expressed.

### Auditory tests

ABRs and DPOAEs were recorded using the BioSigRZ system (Tucker-Davis Technologies, Alachua, FL, USA) in a soundproof chamber. Mice were anesthetized with intraperitoneal injection of xylazine (10 mg/kg) and ketamine (100 mg/kg). Stimuli were generated by digital input/out cards (PXI-4461 National Instruments) in a PXI-1042Q chassis, amplified by a SA-1 speaker driver (Tucker–Davis Technologies, Inc.) and delivered to the studied ear by two electrostatic drivers (CUI Miniature Dynamics). An electret microphone (Electret Condenser) was used to record ear-canal sound pressure. ABR signals were collected using subcutaneous needle electrodes inserted at the pinna (active electrode), vertex (reference electrode), and rump (ground electrode).

Clicks were square pulses 100 ms in duration, and tone bursts were 3 ms in length at distinct 4, 8, 16, 24, and 32 kHz frequencies. The sound level was raised in 5 dB steps from 20 dB below threshold up to 90 dB, and electrical signals were averaged over 512 repeats. The ABR threshold was defined visually as the lowest sound pressure level (dB SPL) at which any wave could be detected. ABR thresholds were averaged within each experimental group and used for statistical analysis. Wave 1 amplitude was defined as the difference between the Wave 1 peak (P1) and the average of the 1 ms pre-stimulus baseline. DPOAE data were collected and recorded under the same conditions as ABR. Primary tones were produced at a frequency ratio of 1.2 (f2/f1) for the generation of DPOAEs at 2f1–f2, where the f2 level was 10 dB SPL below the f1 level for each f2/f1 pair. The f2 levels were swept in 5 dB steps from 20 to 80 dB. Waveform and spectral averaging were used at each level to increase the signal-to-noise ratio of the recorded ear-canal sound pressure. At each level, the amplitude of the DPOAE at 2f1–f2 was extracted from the averaged spectra, along with the noise floor. Iso-response curves were interpolated from plots of DPOAE amplitude versus sound level. The threshold was defined as the f2 level required to produce DPOAEs above 0 dB.

### Immunohistochemistry of the cochlea

Injected and contralateral non-injected cochleae of 10-week-old adult mice were harvested after the animals were sacrificed by cervical dislocation. The temporal bones were perforated on the top of the cochlea and perfused with 4% paraformaldehyde, incubated at 4 °C overnight, and decalcified in 10% EDTA at 4 °C for 1–3 days. The decalcified cochleae were dissected in pieces in PBS for whole-mount immunofluorescence staining. Tissues were permeabilized and infiltrated with 1% Triton X-100 and blocked with 10% donkey serum at 4 °C for 12–16 h and then incubated with rabbit anti-MYO7A primary antibody (#25-6790, Proteus BioSciences, 1:800 dilution) at 4 °C overnight. The secondary antibody Cy3-conjugated donkey anti-rabbit IgG (donkey anti-rabbit AlexJackson ImmunoResearch 711-165-152, 1:500 dilution) was incubated in the dark for 2 h at room temperature after three rinses with PBS. For 2-week-old mice, the cochleae were harvested and dissected without decalcification, and chicken anti-EGFP primary antibody (Abcam, 1:1,000 dilution) was used as the primary antibody to detect the transduction efficiency of AAVs following the same procedure as above. Specimens were mounted on adhesion microscope slides (#188105, Citotest), and confocal images were acquired with a Leica TCS SP8 microscope using a 40× glycerin immersion lens.

### Hair cell counting

ImageJ was used to merge z-stacks by maximum intensity projections of z-stacks for each segment. MYO7A-positive IHCs and OHCs were counted in 100 µm cochlear sections in the 8 and 16 kHz region, approximate frequencies sensed by each region were determined according to the instructions.^20^ The outer three rows of arranged cells were OHCs and the inner single row of were IHCs.

### Scanning electron microscopy

The temporal bones of 10-week-old adult mice were harvested after the animals were sacrificed by cervical dislocation. The temporal bones were perforated and perfused with 2.5% glutaraldehyde on the top of cochlea, fixed in 2.5% glutaraldehyde at 4 °C overnight, and decalcified in 10% EDTA at 4 °C for 3–5 days. The decalcified cochleae were dissected in pieces in 0.1 M PB for whole mounts. The dissected tissues were placed back into 2.5% glutaraldehyde, washed three times with 0.1 M PB, and fixed in 1% osmium acid at 4 °C for 2 h. The tissues were dehydrated in an ethanol gradient and then dried in an HCP-2 critical point dryer for 2 h. The dried tissues were pasted on the sample tables and sprayed with an IB-3 ion sputtering instrument for 3 min. SEM images were acquired at 2.5 kV (low magnification) or 10.0 kV (high magnification) with a high vacuum field emission SEM (Hitachi SU-8010).

### Statistics

All data are shown as the mean ± SD. Statistical analysis of the results was performed using GraphPad Prism (GraphPad PRISM, Version 8.0). Student’s t-test was used to determine significant differences between the means, and one-way analysis of variance (ANOVA) or two-way ANOVA was used for multiple comparisons. The level of significance was set at *P* < 0.05.

## Supplementary information

Supplementary materials

Dataset 1

Dataset 2

Dataset 3

## Data Availability

The data supporting the finding of this study are available within the article and its supplementary Information files or available from the corresponding author on reasonable request.
