# Supplementary material for: Preventing autosomal-dominant hearing loss in Bth mice with CRISPR/CasRx-based RNA editing
Source: Signal Transduct Target Ther. 2022 Mar 14;7:79. doi: 10.1038/s41392-022-00893-4 (PMC8918553; doi:10.1038/s41392-022-00893-4)
Supplement: Supplementary file 1 — Supplementary materials [file 41392_2022_893_MOESM1_ESM.docx]

**Supplementary materials**

**Preventing autosomal-dominant hearing loss in *Bth* mice with CRISPR/CasRx-based RNA editing**

**Authors:** Ziwen Zheng^1,2,3^, Guo Li^4,5^, Chong Cui^1,2,3^, Fang Wang^1,2,3^, Xiaohan Wang^6^, Zhijiao Xu^1,2,3^, Huiping Guo^1,2,3^, Yuxin Chen^1,2,3^, Honghai Tang^1,2,3^, Daqi Wang^1,2,3^, Mingqian Huang^7,8^, Zheng-Yi Chen^7,8^, Xingxu Huang^9,10^, Huawei Li^1,2,3,11^, Geng-Lin Li^1,2,3^, Xiaoxiang Hu^4,5^, Yilai Shu^1,2,3^

**AFFILIATIONS:**

^1^ENT institute and Department of Otorhinolaryngology, Eye & ENT Hospital, State Key Laboratory of Medical Neurobiology and MOE Frontiers Center for Brain Science, Fudan University, Shanghai, 200031, China. ^2^Institutes of Biomedical Sciences, Fudan University, Shanghai, 200032, China. ^3^NHC Key Laboratory of Hearing Medicine (Fudan University), Shanghai, 200032, China. ^4^State Key Laboratory of Agrobiotechnology, China Agricultural University, Beijing 100193, China. ^5^College of Biological Sciences, China Agricultural University, Beijing, 100193, China. ^6^School of Life Science and Technology, Southeast University, Nanjing 210096, China. ^7^Department of Otolaryngology-Head and Neck Surgery, Graduate Program in Speech and Hearing Bioscience and Technology and Program in Neuroscience, Harvard Medical School, Boston, MA 02115, USA. ^8^Eaton-Peabody Laboratory, Massachusetts Eye and Ear Infirmary, Boston, MA 02114, USA. ^9^School of Life Science and Technology, ShanghaiTech University, Shanghai, 200031, China. ^10^CAS Center for Excellence in Molecular Cell Science, Shanghai Institute of Biochemistry and Cell Biology, Chinese Academy of Sciences, University of Chinese Academy of Sciences, Shanghai, 200031, China. ^11^The Institutes of Brain Science and the Collaborative Innovation Center for Brain Science, Fudan University, Shanghai, 200031, China.

Correspondence: Yilai Shu (yilai_shu@fudan.edu.cn), Xiaoxiang Hu (huxx@cau.edu.cn) or Geng-Lin Li (genglin.li@fdeent.org)

These authors contributed equally: Ziwen Zheng, Guo Li, and Chong Cui

**Supplementary Figure 1.**

**
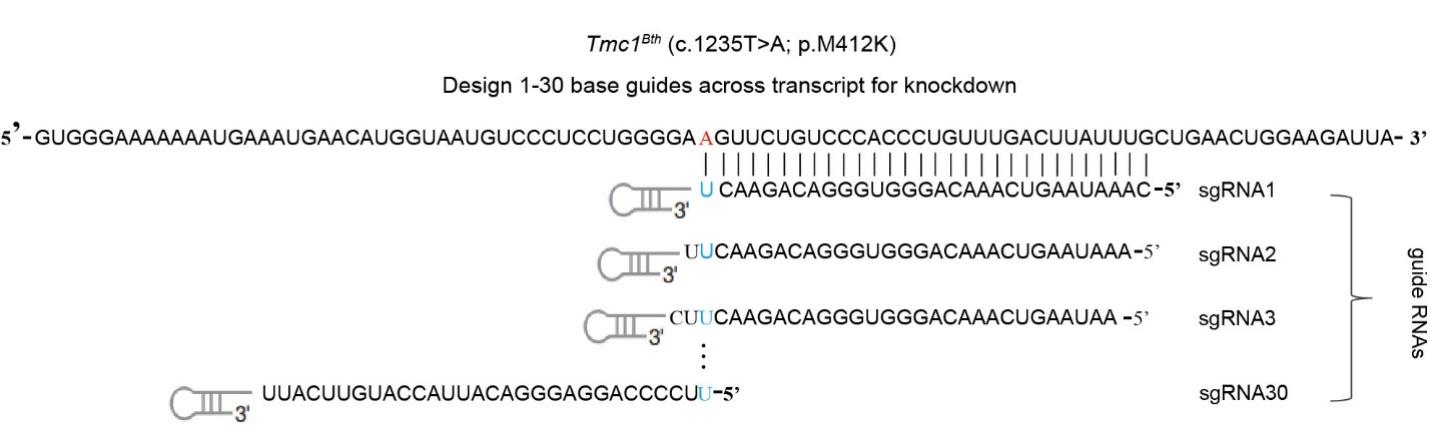
**

**Fig. S1.** Designing of sgRNAs for targeting the *Tmc1^Bth^* transcript. Schematic diagram for designing sgRNAs to target the *Tmc1^Bth^* transcript. A total of 30 sgRNAs were designed, in which the base ‘A’ was inserted at positions 1 to 30 in turn.

**Supplementary Figure 2.**


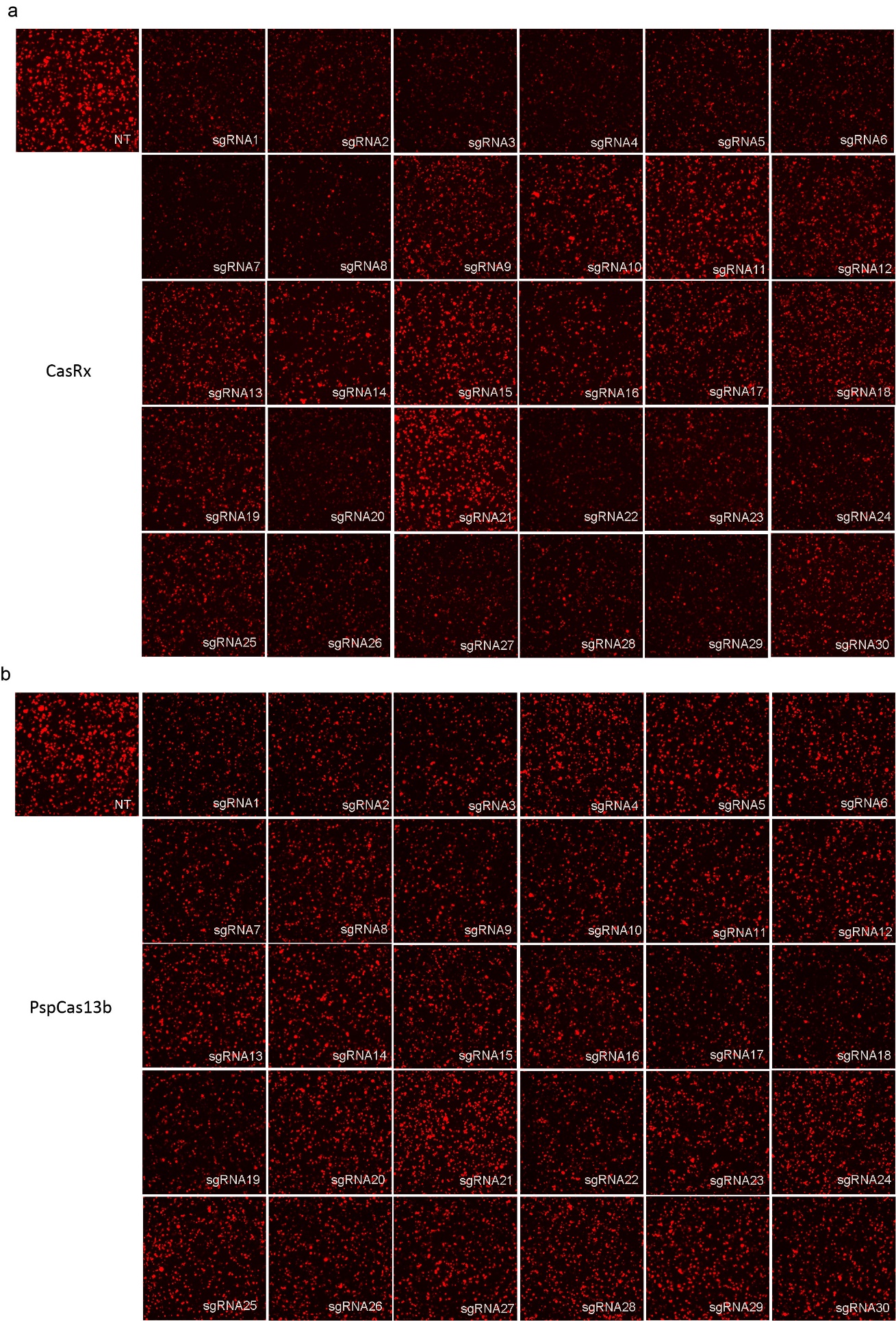


**Fig. S2.** Representative fluorescence images of 293T cells. **a** Cells were transfected with CasRx system with 30 sgRNAs and non-target sgRNA to target *Tmc1^Bth^* transcript. **b** Cells were transfected with PspCas13b system with 30 sgRNAs and non-target sgRNA to target *Tmc1^Bth^* transcript. The mCherry intensities indicated efficiency of RNA knockdown.

**Supplementary Figure 3.**


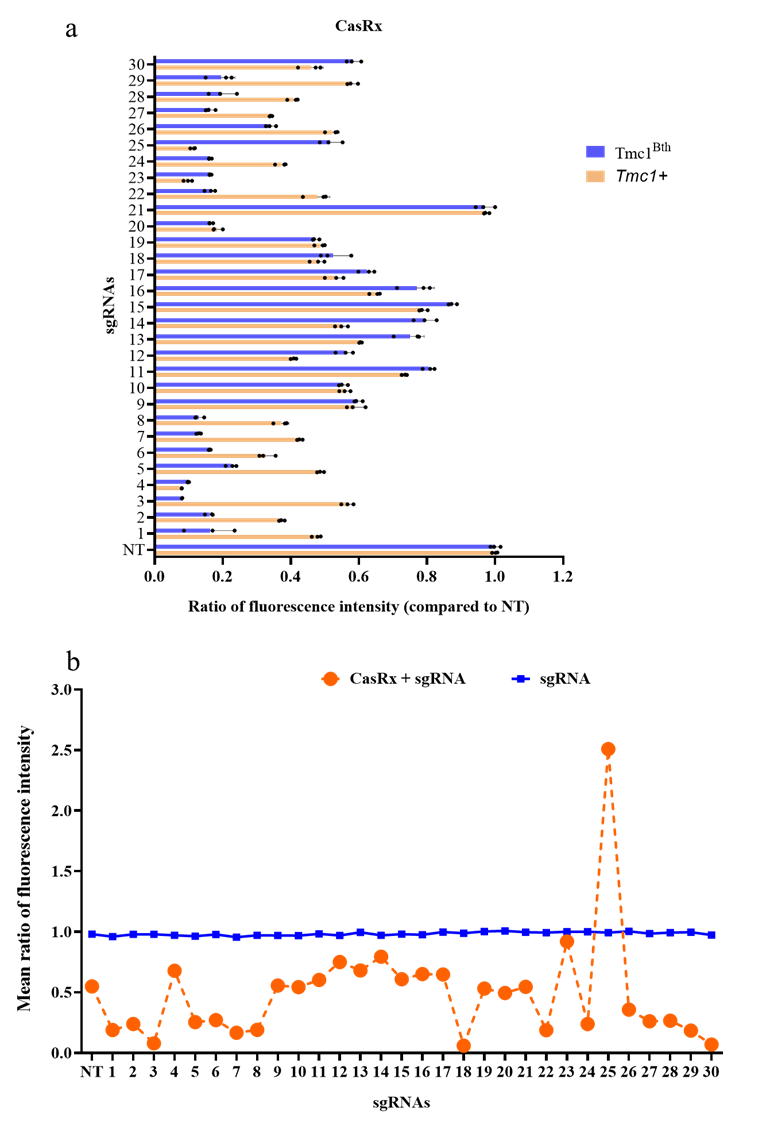


**Fig. S3.** Repeat experiments of cell fluorescence intensities mediated by CasRx. **a** Fluorescence intensities of mCherry-*Tmc1^Bth^* and mCherry-*Tmc1^+^* mRNA in cell cultures with different sgRNAs. **b** Mean ratio of fluorescence intensities between mCherry-*Tmc1^Bth^* and mCherry-*Tmc1^+^* mRNA.

**Supplementary Figure 4.**


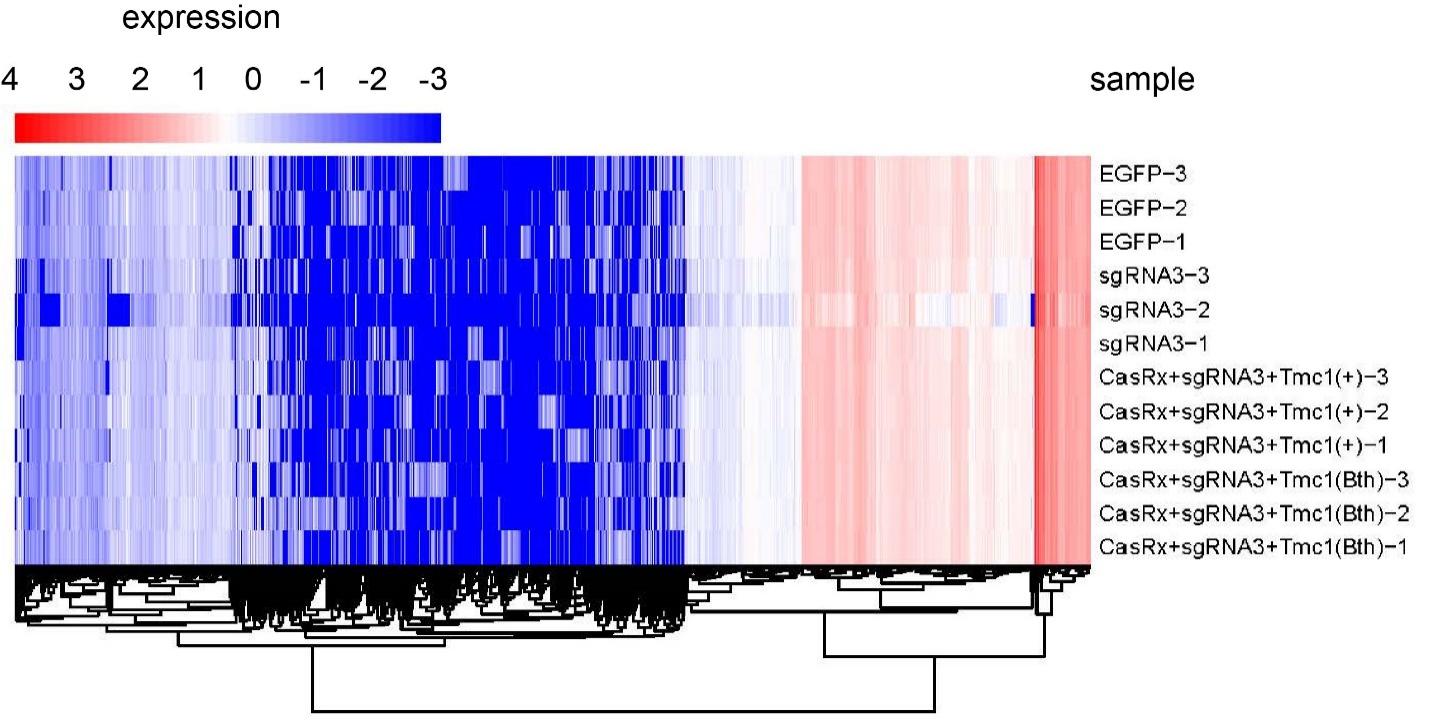


**Fig. S4.** FPKM heatmap of all the expressed genes in 293T cells, created with the gplots package (version 3.0.1.1.) in R (version 3.6.1).

**Supplementary Figure 5.**


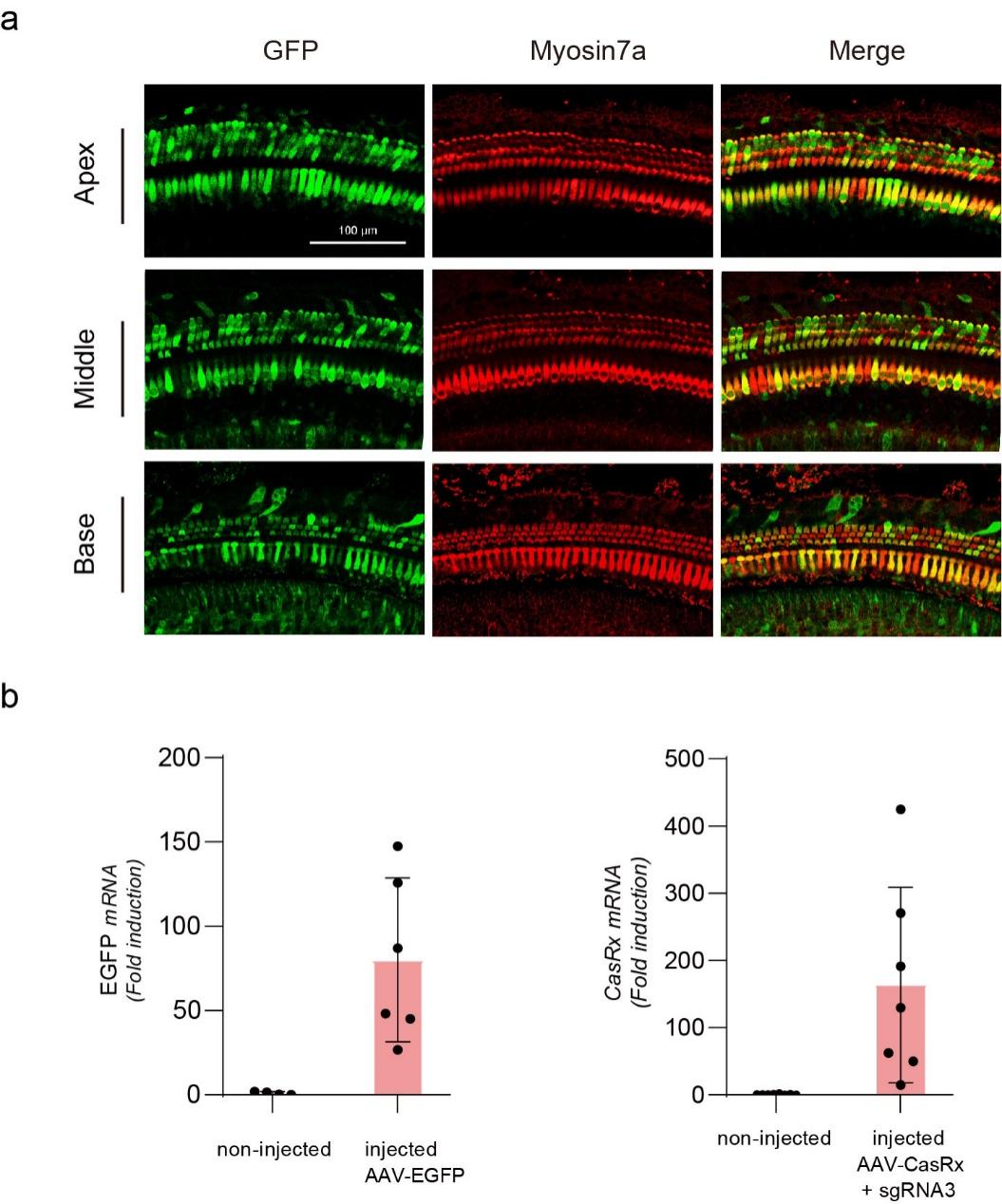


**Fig. S5.** Transduction test of AAV-PHP.eB. Cochleae were harvested 2 weeks after injection. **a** Samples were stained with anti-GFP and myosin7a antibodies, and images represent 300 μm lengths of whole-mount cochlear tissue. Scale bar: 100 μm. **b**  The expression of CasRx (n = 7 mice) and EGFP mRNA (n = 6 mice). Cochleae injected AAV compared to cochleae from non-injected mice.

**Supplementary Figure 6.**


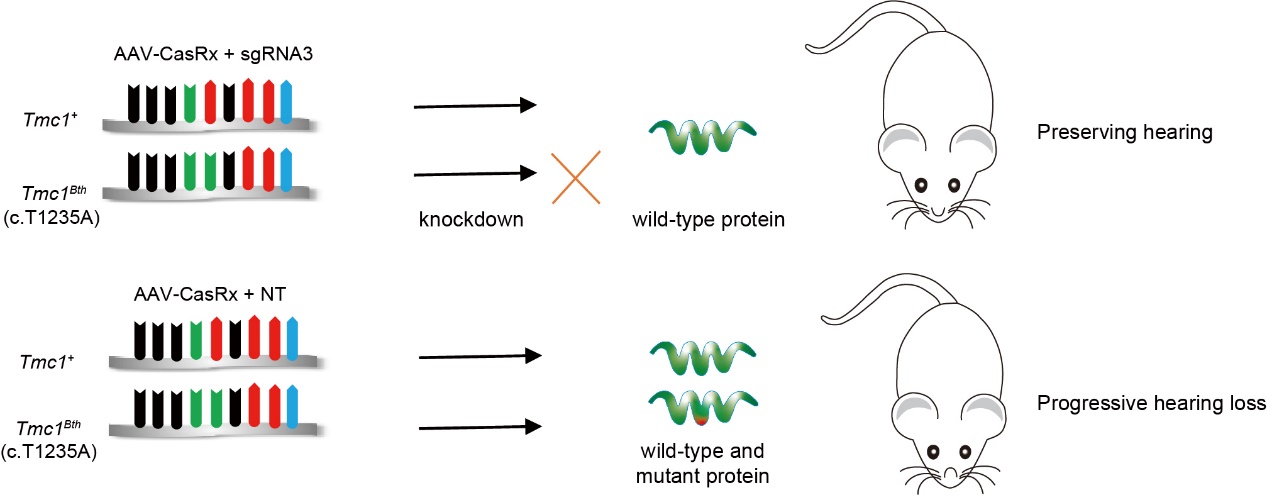


**Fig. S6.** Improvement in hearing by mRNA knockdown. Schematic overview with and without CasRx-mediated gene silencing in *Tmc1^Bth/+^* mice to prevent progressive hearing loss.

**Supplementary Figure 7.**


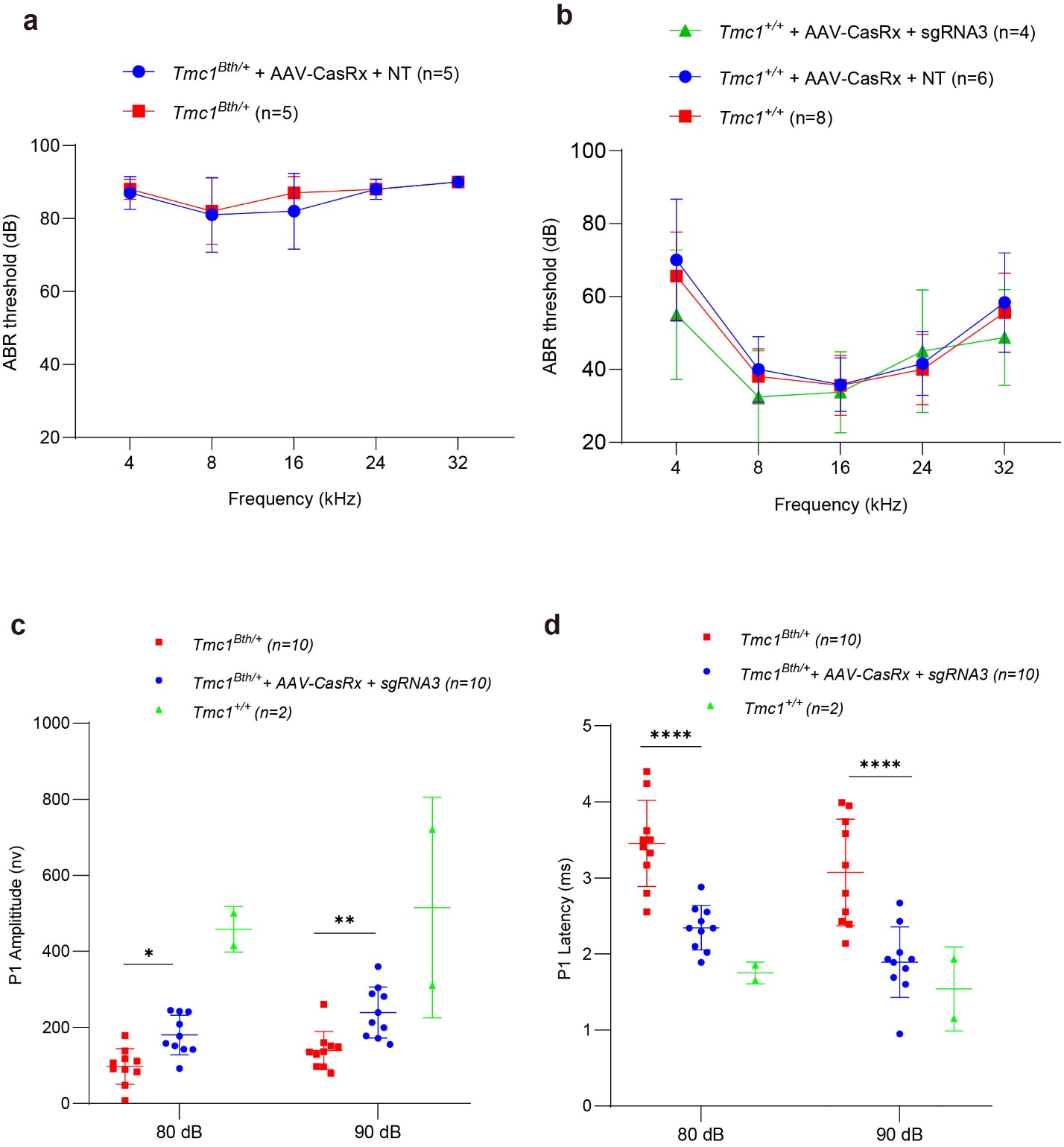


**Fig. S7.** ABR of mouse injected with AAV. **a** The thresholds of the heterogeneous mouse ears injected with control AAV and non-injected contralateral ears at 4 weeks of age. **b** The thresholds of wild-type mouse ears injected with AAV (AAV-CasRx + sgRNA3 or AAV-CasRx + NT) and non-injected contralateral ears at 4 weeks of age. Data are shown as the mean ± SD. **c, d** Click-ABR P1 amplitudes and latencies of wave 1 at 80 dB and 90 dB in AAV-CasRx + sgRNA3 injected, non-injected *Bth* mice, and wild-type mice at 4 weeks (**P* < 0.05, ***P* < 0.01, *****P* < 0.0001). Mean ± SD, statistical analysis was performed by Tukey's multiple comparisons test.

**Supplementary Figure 8.**


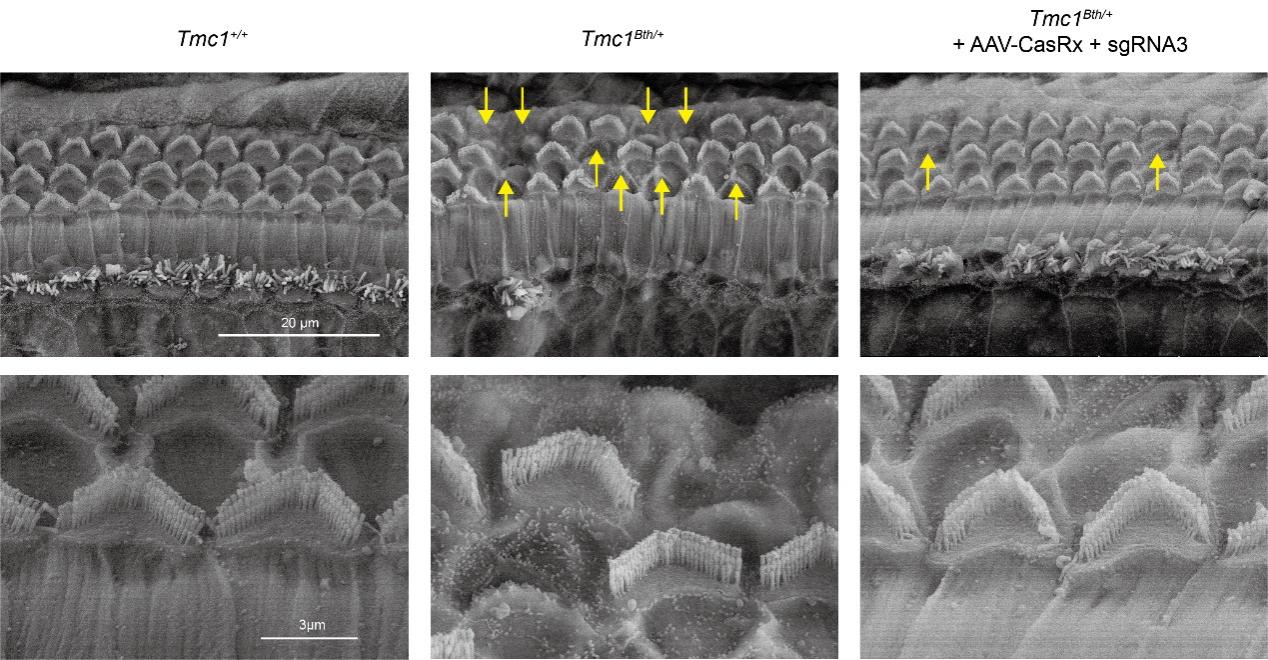


**Fig. S8.** SEM images of the middle cochlear sensory epithelium showing the morphology of hair cell bundles. *Tmc1*^+/+^, *Tmc1^Bth/+^* + AAV-CasRx + sgRNA3, and *Tmc1^Bth/+^* non-injected samples were collected 10 weeks after injection. Arrows show deficiency of hair cells bundles. Scale bars: 20 μm (upper); 3 μm (lower).

**Supplementary Table 1.** Sequences of sgRNA used in RNA editing expreiments.

| sgRNA1 | 5’-CAA ATA AGT CAA ACA GGG TGG GAC AGA ACT-3’ |
| --- | --- |
| sgRNA2 | 5’-AAA TAA GTC AAA CAG GGT GGG ACA GAA CTT-3’ |
| sgRNA3 | 5’-AAT AAG TCA AAC AGG GTG GGA CAG AAC TTC-3’ |
| sgRNA4 | 5’-ATA AGT CAA ACA GGG TGG GAC AGA ACT TCC-3’ |
| sgRNA5 | 5’-TAA GTC AAA CAG GGT GGG ACA GAA CTT CCC-3’ |
| sgRNA6 | 5’-AAG TCA AAC AGG GTG GGA CAG AAC TTC CCC-3’ |
| sgRNA7 | 5’-AGT CAA ACA GGG TGG GAC AGA ACT TCC CCA-3’ |
| sgRNA8 | 5’-GTC AAA CAG GGT GGG ACA GAA CTT CCC CAG-3’ |
| sgRNA9 | 5’-TCA AAC AGG GTG GGA CAG AAC TTC CCC AGG-3’ |
| sgRNA10 | 5’-CAA ACA GGG TGG GAC AGA ACT TCC CCA GGA-3’ |
| sgRNA11 | 5’-AAA CAG GGT GGG ACA GAA CTT CCC CAG GAG-3’ |
| sgRNA12 | 5’-AAC AGG GTG GGA CAG AAC TTC CCC AGG AGG-3’ |
| sgRNA13 | 5’-ACA GGG TGG GAC AGA ACT TCC CCA GGA GGG-3’ |
| sgRNA14 | 5’-CAG GGT GGG ACA GAA CTT CCC CAG GAG GGA-3’ |
| sgRNA15 | 5’-AGG GTG GGA CAG AAC TTC CCC AGG AGG GAC-3’ |
| sgRNA16 | 5’-GGG TGG GAC AGA ACT TCC CCA GGA GGG ACA-3’ |
| sgRNA17 | 5’-GGT GGG ACA GAA CTT CCC CAG GAG GGA CAT-3’ |
| sgRNA18 | 5’-GTG GGA CAG AAC TTC CCC AGG AGG GAC ATT-3’ |
| sgRNA19 | 5’-TGG GAC AGA ACT TCC CCA GGA GGG ACA TTA-3’ |
| sgRNA20 | 5’-GGG ACA GAA CTT CCC CAG GAG GGA CAT TAC-3’ |
| sgRNA21 | 5’-GGA CAG AAC TTC CCC AGG AGG GAC ATT ACC-3’ |
| sgRNA22 | 5’-GAC AGA ACT TCC CCA GGA GGG ACA TTA CCA-3’ |
| sgRNA23 | 5’-ACA GAA CTT CCC CAG GAG GGA CAT TAC CAT-3’ |
| sgRNA24 | 5’-CAG AAC TTC CCC AGG AGG GAC ATT ACC ATG-3’ |
| sgRNA25 | 5’-AGA ACT TCC CCA GGA GGG ACA TTA CCA TGT-3’ |
| sgRNA26 | 5’-GAA CTT CCC CAG GAG GGA CAT TAC CAT GTT-3’ |
| sgRNA27 | 5’-AAC TTC CCC AGG AGG GAC ATT ACC ATG TTC-3’ |
| sgRNA28 | 5’-ACT TCC CCA GGA GGG ACA TTA CCA TGT TCA-3’ |
| sgRNA29 | 5’-CTT CCC CAG GAG GGA CAT TAC CAT GTT CAT-3’ |
| sgRNA30 | 5’-TTC CCC AGG AGG GAC ATT ACC ATG TTC ATT-3’ |
| NT | 5’-ACC AGG ATG GGC ACC ACC CCG GTG AAC AGC-3’ |

| mCherry expression backbone | F: 5’-TGA ACT GGA AGA TTA AAG CTT AAG TTT AAA CCG CT-3’;  R: 5’-TCA TTT TTT TCC CAC CTT GTA CAG CTC GTC CAT GC-3’ |
| --- | --- |
| *Tmc1^Bth^* | F: 5’-GTG GGA AAA AAA TGA AAT GAA CAT GGT AAT GTC CCT CCT GGG GAA GTT CTG TCC CAC CCT GTT TGA CTT ATT TGC TGA ACT GGA AGA TTA-3’;  R: 5’-TAA TCT TCC AGT TCA GCA AAT AAG TCA AAC AGG GTG GGA CAG AAC TTC CCC AGG AGG GAC ATT ACC ATG TTC ATT TCA TTT TTT TCC CAC-3’ |
| *Tmc1*^+^ | F: 5’-GTG GGA AAA AAA TGA AAT GAA CAT GGT AAT GTC CCT CCT GGG GAT GTT CTG TCC CAC CCT GTT TGA CTT ATT TGC TGA ACT GGA AGA TTA-3’;  R: 5’-TAA TCT TCC AGT TCA GCA AAT AAG TCA AAC AGG GTG GGA CAG AAC ATC CCC AGG AGG GAC ATT ACC ATG TTC ATT TCA TTT TTT TCC CAC-3’ |
| Genotyping | F: 5’-CTA ATC ATA CCA AGG AAA CAT ATG GAC-3’;  R: 5’-TAG ACT CAC CTT GTT GTT AAT CTC ATC-3’ |
| Deep sequencing | TMC1-lib F: 5’-ACA CTC TTT CCC TAC ACG ACG CTC TTC CGA TCT NNN NGT GGA TAC CTC ATC TTT TGG G-3’;  TMC1-lib R: 5’-ACT GGA GTT CAG ACG TGT GCT CTT CCG ATC TGC CAC TTC AGA GCA ATG AGA G-3’ |
| RT-qPCR | q-TMC1-F2: 5’-AAT GAA CAT GGT AAT GTC CC-3’;  q-TMC1-R2: 5’-CTC CTC TTC AAT CTT GTT GT-3’;  q-TMC1-F4: 5’-TAA TGT CCC TCC TGG GGA A-3’  q-TMC1-F5: 5’-TAA TGT CCC TCC TGG GGA T-3’  q-mouse actin-f: 5’-GAG ACC TTC AAC ACC CCA GC-3’  q-mouse actin-r: 5’-ATG TCA CGC ACG ATT TCC C-3’  q-egfp-f: 5’-GGA CGA CGG CAA CTA CAA GA-3’  q-egfp-r: 5’-AAG TCG ATG CCC TTC AGC TC-3’  q-CasRx-f: 5’-AAG GGC TTT AGC TCC GTG AC-3’  q-CasRx-r: 5’-GTT GGC GTA CAG CTT AGG GT5’- |

**Supplementary Table 2.** Primers for plasmids construction, genotyping, deep sequencing, and RT-PCR.

**Supplementary Table 3.** Deep sequencing data of cochlear samples. Samples (n = 3 mice) were injected, non-injected or injected non-targeted virus**.**

| Samples | Total reads | Wt reads | Mut reads | Mut ratio | Background |
| --- | --- | --- | --- | --- | --- |
| HE-T-1 | 628,899 | 457,113 | 161,862 | 0.261500061 | 0.015780 |
| HE-T-2 | 693,555 | 623,612 | 59,743 | 0.087426008 | 0.014707 |
| HE-T-4 | 646,412 | 573,649 | 61,993 | 0.097528168 | 0.016661 |
| Het-CT-1 | 740,710 | 307,819 | 421,008 | 0.577651487 | 0.016043 |
| Het-CT-2 | 979,293 | 425,342 | 502,410 | 0.541534807 | 0.052631 |
| Het-CT-3 | 1,128,677 | 571,231 | 532,729 | 0.482561868 | 0.021899 |
| HE-ut-1 | 1,943,326 | 781,915 | 1,123,868 | 0.589714569 | 0.019319 |
| HE-ut-2 | 571,456 | 283,888 | 276,354 | 0.49327612 | 0.019624 |
| HE-ut-3 | 801,693 | 391,520 | 394,741 | 0.502048302 | 0.019249 |

|  | Gene name | Gene_chr | Gene_description |
| --- | --- | --- | --- |
| Off-target-1 | *TMC1* | 9 | transmembrane channel like 1[Source:HGNC Symbol;Acc: HGNC:16513 |
| Off-target-2 | *ACSF2* | 17 | acyl-CoA synthetase family member 2 [Source:HGNC Symbol;Acc:HGNC:26101] |
| Off-target-3 | *KCNB1* | 20 | potassium voltage-gated channel subfamily B member 1 [Source:HGNC Symbol;Acc:HGNC:6231] |
| Off-target-4 | *RXRA* | 9 | retinoid X receptor alpha [Source:HGNC Symbol;Acc:HGNC:10477] |
| Off-target-5 | *AC092546.1* |  | Homo sapiens chromosome RPCI-11 clone RP11-801O14, WORKING DRAFT SEQUENCE, 26 unordered pieces. |
| Off-target-6 | *EFCAB6* | 22 | EF-hand calcium binding domain 6 [Source:HGNC Symbol;Acc:HGNC:24204] |
| Off-target-7 | *ANO1* | 11 | anoctamin 1 [Source:HGNC Symbol;Acc:HGNC:21625] |
| Off-target-8 | *DGCR2* | 22 | DiGeorge syndrome critical region gene 2 [Source:HGNC Symbol;Acc:HGNC:2845] |
| Off-target-9 | *BIK* | 22 | BCL2 interacting killer [Source:HGNC Symbol;Acc:HGNC:1051] |
| Off-target-10 | *TNFRSF8* | 1 | TNF receptor superfamily member 8 [Source:HGNC Symbol;Acc:HGNC:11923] |

**Supplementary Table 4.** **Off-target sites.**

The top 10 most likely off-target genes screened by aligning with sequence of sgRNA3 in 293T cells.

|  | Gene name | Gene_chr | Gene_description |
| --- | --- | --- | --- |
| Off-target-1 | *Tnrc6c* | 11 | Trinucleotide repeat containing 6C [Source:MGI Symbol;Acc:MGI:2443265] |
| Off-target-2 | *Ctbs* | 3 | Chitobiase [Source:MGI Symbol;Acc:MGI:1921495] |
| Off-target-3 | *Cnot4* | 6 | CCR4-NOT transcription complex, subunit 4 [Source:MGI Symbol;Acc:MGI:1859026] |
| Off-target-4 | *Gm13492* | 2 | Predicted gene 13492 [Source:MGI Symbol;Acc:MGI:3650823] |
| Off-target-5 | *Amdhd2* | 17 | Amidohydrolase domain containing 2 [Source:MGI Symbol;Acc:MGI:2443978] |
| Off-target-6 | *G2e3* | 12 | G2/M-phase specific E3 ubiquitin ligase [Source:MGI Symbol;Acc:MGI:2444298] |
| Off-target-7 | *Sgsh* | 11 | N-sulfoglucosamine sulfohydrolase (sulfamidase) [Source:MGI Symbol;Acc:MGI:1350341] |
| Off-target-8 | *Mbd1* | 18 | Methyl-CpG binding domain protein 1 [Source:MGI Symbol;Acc:MGI:1333811] |
| Off-target-9 | *Mcemp1* | 8 | Mast cell expressed membrane protein 1 [Source:MGI Symbol;Acc:MGI:1916439] |
| Off-target-10 | *Aldh8a1* | 10 | Aldehyde dehydrogenase 8 family, member A1 [Source:MGI Symbol;Acc:MGI:2653900] |

The top 10 most likely off-target genes screened by aligning with sequence of sgRNA3 *in vivo*.

**Expression vectors**

(1) Fluorescence reporters.

mCherry and *Tmc1* targets are highlighted in red and blue, respectively. The start codon is capitalized.

**mCherry-*Tmc1*-mut:**

GACGGATCGGGAGATCTCCCGATCCCCTATGGTGCACTCTCAGTACAATCTGCTCTGATGCCGCATAGTTAAGCCAGTATCTGCTCCCTGCTTGTGTGTTGGAGGTCGCTGAGTAGTGCGCGAGCAAAATTTAAGCTACAACAAGGCAAGGCTTGACCGACAATTGCATGAAGAATCTGCTTAGGGTTAGGCGTTTTGCGCTGCTTCGCGATGTACGGGCCAGATATACGCGTTGACATTGATTATTGACTAGTTATTAATAGTAATCAATTACGGGGTCATTAGTTCATAGCCCATATATGGAGTTCCGCGTTACATAACTTACGGTAAATGGCCCGCCTGGCTGACCGCCCAACGACCCCCGCCCATTGACGTCAATAATGACGTATGTTCCCATAGTAACGCCAATAGGGACTTTCCATTGACGTCAATGGGTGGAGTATTTACGGTAAACTGCCCACTTGGCAGTACATCAAGTGTATCATATGCCAAGTACGCCCCCTATTGACGTCAATGACGGTAAATGGCCCGCCTGGCATTATGCCCAGTACATGACCTTATGGGACTTTCCTACTTGGCAGTACATCTACGTATTAGTCATCGCTATTACCATGGTGATGCGGTTTTGGCAGTACATCAATGGGCGTGGATAGCGGTTTGACTCACGGGGATTTCCAAGTCTCCACCCCATTGACGTCAATGGGAGTTTGTTTTGGCACCAAAATCAACGGGACTTTCCAAAATGTCGTAACAACTCCGCCCCATTGACGCAAATGGGCGGTAGGCGTGTACGGTGGGAGGTCTATATAAGCAGAGCTCTCTGGCTAACTAGAGAACCCACTGCTTACTGGCTTATCGAAATTAATACGACTCACTATAGGGAGACCCAAGCTGGCTAGCGTTTAAACGGGCCCTCTAGACTCGAG**ATG**GTGAGCAAGGGCGAGGAGGATAACATGGCCATCATCAAGGAGTTCATGCGCTTCAAGGTGCACATGGAGGGCTCCGTGAACGGCCACGAGTTCGAGATCGAGGGCGAGGGCGAGGGCCGCCCCTACGAGGGCACCCAGACCGCCAAGCTGAAGGTGACCAAGGGTGGCCCCCTGCCCTTCGCCTGGGACATCCTGTCCCCTCAGTTCATGTACGGCTCCAAGGCCTACGTGAAGCACCCCGCCGACATCCCCGACTACTTGAAGCTGTCCTTCCCCGAGGGCTTCAAGTGGGAGCGCGTGATGAACTTCGAGGACGGCGGCGTGGTGACCGTGACCCAGGACTCCTCCCTGCAGGACGGCGAGTTCATCTACAAGGTGAAGCTGCGCGGCACCAACTTCCCCTCCGACGGCCCCGTAATGCAGAAGAAGACCATGGGCTGGGAGGCCTCCTCCGAGCGGATGTACCCCGAGGACGGCGCCCTGAAGGGCGAGATCAAGCAGAGGCTGAAGCTGAAGGACGGCGGCCACTACGACGCTGAGGTCAAGACCACCTACAAGGCCAAGAAGCCCGTGCAGCTGCCCGGCGCCTACAACGTCAACATCAAGTTGGACATCACCTCCCACAACGAGGACTACACCATCGTGGAACAGTACGAACGCGCCGAGGGCCGCCACTCCACCGGCGGCATGGACGAGCTGTACAAGAGTGGATACCTCATCTTTTGGGCTGTGAAGCGATCCCAGGAGTTCGCCCAGCAAGATCCTGACACCCTTGGGTGGTGGGAAAAAAATGAAATGAACATGGTAATGTCCCTCCTGGGGAAGTTCTGTCCCACCCTGTTTGACTTATTTGCTGAACTGGAAGATTACCATCCTCTCATTGCTCTGAAGTGGCTCCTGGGGCGCATTTTTGCTCTTCTTCTAGGCAACTTGTATGTATTCATTCTCGCCTTGATGGATGAGATTAACAACAAGATTGAAGAGGAGAAGCTTGTGAAGGCCAATAAGCTTAAGTTTAAACCGCTGATCAGCCTCGACTGTGCCTTCTAGTTGCCAGCCATCTGTTGTTTGCCCCTCCCCCGTGCCTTCCTTGACCCTGGAAGGTGCCACTCCCACTGTCCTTTCCTAATAAAATGAGGAAATTGCATCGCATTGTCTGAGTAGGTGTCATTCTATTCTGGGGGGTGGGGTGGGGCAGGACAGCAAGGGGGAGGATTGGGAAGACAATAGCAGGCATGCTGGGGATGCGGTGGGCTCTATGGCTTCTGAGGCGGAAAGAACCAGCTGGGGCTCTAGGGGGTATCCCCACGCGCCCTGTAGCGGCGCATTAAGCGCGGCGGGTGTGGTGGTTACGCGCAGCGTGACCGCTACACTTGCCAGCGCCCTAGCGCCCGCTCCTTTCGCTTTCTTCCCTTCCTTTCTCGCCACGTTCGCCGGCTTTCCCCGTCAAGCTCTAAATCGGGGGCTCCCTTTAGGGTTCCGATTTAGTGCTTTACGGCACCTCGACCCCAAAAAACTTGATTAGGGTGATGGTTCACGTAGTGGGCCATCGCCCTGATAGACGGTTTTTCGCCCTTTGACGTTGGAGTCCACGTTCTTTAATAGTGGACTCTTGTTCCAAACTGGAACAACACTCAACCCTATCTCGGTCTATTCTTTTGATTTATAAGGGATTTTGCCGATTTCGGCCTATTGGTTAAAAAATGAGCTGATTTAACAAAAATTTAACGCGAATTAATTCTGTGGAATGTGTGTCAGTTAGGGTGTGGAAAGTCCCCAGGCTCCCCAGCAGGCAGAAGTATGCAAAGCATGCATCTCAATTAGTCAGCAACCAGGTGTGGAAAGTCCCCAGGCTCCCCAGCAGGCAGAAGTATGCAAAGCATGCATCTCAATTAGTCAGCAACCATAGTCCCGCCCCTAACTCCGCCCATCCCGCCCCTAACTCCGCCCAGTTCCGCCCATTCTCCGCCCCATGGCTGACTAATTTTTTTTATTTATGCAGAGGCCGAGGCCGCCTCTGCCTCTGAGCTATTCCAGAAGTAGTGAGGAGGCTTTTTTGGAGGCCTAGGCTTTTGCAAAAAGCTCCCGGGAGCTTGTATATCCATTTTCGGATCTGATCAAGAGACAGGATGAGGATCGTTTCGCATGATTGAACAAGATGGATTGCACGCAGGTTCTCCGGCCGCTTGGGTGGAGAGGCTATTCGGCTATGACTGGGCACAACAGACAATCGGCTGCTCTGATGCCGCCGTGTTCCGGCTGTCAGCGCAGGGGCGCCCGGTTCTTTTTGTCAAGACCGACCTGTCCGGTGCCCTGAATGAACTGCAGGACGAGGCAGCGCGGCTATCGTGGCTGGCCACGACGGGCGTTCCTTGCGCAGCTGTGCTCGACGTTGTCACTGAAGCGGGAAGGGACTGGCTGCTATTGGGCGAAGTGCCGGGGCAGGATCTCCTGTCATCTCACCTTGCTCCTGCCGAGAAAGTATCCATCATGGCTGATGCAATGCGGCGGCTGCATACGCTTGATCCGGCTACCTGCCCATTCGACCACCAAGCGAAACATCGCATCGAGCGAGCACGTACTCGGATGGAAGCCGGTCTTGTCGATCAGGATGATCTGGACGAAGAGCATCAGGGGCTCGCGCCAGCCGAACTGTTCGCCAGGCTCAAGGCGCGCATGCCCGACGGCGAGGATCTCGTCGTGACCCATGGCGATGCCTGCTTGCCGAATATCATGGTGGAAAATGGCCGCTTTTCTGGATTCATCGACTGTGGCCGGCTGGGTGTGGCGGACCGCTATCAGGACATAGCGTTGGCTACCCGTGATATTGCTGAAGAGCTTGGCGGCGAATGGGCTGACCGCTTCCTCGTGCTTTACGGTATCGCCGCTCCCGATTCGCAGCGCATCGCCTTCTATCGCCTTCTTGACGAGTTCTTCTGAGCGGGACTCTGGGGTTCGAAATGACCGACCAAGCGACGCCCAACCTGCCATCACGAGATTTCGATTCCACCGCCGCCTTCTATGAAAGGTTGGGCTTCGGAATCGTTTTCCGGGACGCCGGCTGGATGATCCTCCAGCGCGGGGATCTCATGCTGGAGTTCTTCGCCCACCCCAACTTGTTTATTGCAGCTTATAATGGTTACAAATAAAGCAATAGCATCACAAATTTCACAAATAAAGCATTTTTTTCACTGCATTCTAGTTGTGGTTTGTCCAAACTCATCAATGTATCTTATCATGTCTGTATACCGTCGACCTCTAGCTAGAGCTTGGCGTAATCATGGTCATAGCTGTTTCCTGTGTGAAATTGTTATCCGCTCACAATTCCACACAACATACGAGCCGGAAGCATAAAGTGTAAAGCCTGGGGTGCCTAATGAGTGAGCTAACTCACATTAATTGCGTTGCGCTCACTGCCCGCTTTCCAGTCGGGAAACCTGTCGTGCCAGCTGCATTAATGAATCGGCCAACGCGCGGGGAGAGGCGGTTTGCGTATTGGGCGCTCTTCCGCTTCCTCGCTCACTGACTCGCTGCGCTCGGTCGTTCGGCTGCGGCGAGCGGTATCAGCTCACTCAAAGGCGGTAATACGGTTATCCACAGAATCAGGGGATAACGCAGGAAAGAACATGTGAGCAAAAGGCCAGCAAAAGGCCAGGAACCGTAAAAAGGCCGCGTTGCTGGCGTTTTTCCATAGGCTCCGCCCCCCTGACGAGCATCACAAAAATCGACGCTCAAGTCAGAGGTGGCGAAACCCGACAGGACTATAAAGATACCAGGCGTTTCCCCCTGGAAGCTCCCTCGTGCGCTCTCCTGTTCCGACCCTGCCGCTTACCGGATACCTGTCCGCCTTTCTCCCTTCGGGAAGCGTGGCGCTTTCTCATAGCTCACGCTGTAGGTATCTCAGTTCGGTGTAGGTCGTTCGCTCCAAGCTGGGCTGTGTGCACGAACCCCCCGTTCAGCCCGACCGCTGCGCCTTATCCGGTAACTATCGTCTTGAGTCCAACCCGGTAAGACACGACTTATCGCCACTGGCAGCAGCCACTGGTAACAGGATTAGCAGAGCGAGGTATGTAGGCGGTGCTACAGAGTTCTTGAAGTGGTGGCCTAACTACGGCTACACTAGAAGAACAGTATTTGGTATCTGCGCTCTGCTGAAGCCAGTTACCTTCGGAAAAAGAGTTGGTAGCTCTTGATCCGGCAAACAAACCACCGCTGGTAGCGGTTTTTTTGTTTGCAAGCAGCAGATTACGCGCAGAAAAAAAGGATCTCAAGAAGATCCTTTGATCTTTTCTACGGGGTCTGACGCTCAGTGGAACGAAAACTCACGTTAAGGGATTTTGGTCATGAGATTATCAAAAAGGATCTTCACCTAGATCCTTTTAAATTAAAAATGAAGTTTTAAATCAATCTAAAGTATATATGAGTAAACTTGGTCTGACAGTTACCAATGCTTAATCAGTGAGGCACCTATCTCAGCGATCTGTCTATTTCGTTCATCCATAGTTGCCTGACTCCCCGTCGTGTAGATAACTACGATACGGGAGGGCTTACCATCTGGCCCCAGTGCTGCAATGATACCGCGAGACCCACGCTCACCGGCTCCAGATTTATCAGCAATAAACCAGCCAGCCGGAAGGGCCGAGCGCAGAAGTGGTCCTGCAACTTTATCCGCCTCCATCCAGTCTATTAATTGTTGCCGGGAAGCTAGAGTAAGTAGTTCGCCAGTTAATAGTTTGCGCAACGTTGTTGCCATTGCTACAGGCATCGTGGTGTCACGCTCGTCGTTTGGTATGGCTTCATTCAGCTCCGGTTCCCAACGATCAAGGCGAGTTACATGATCCCCCATGTTGTGCAAAAAAGCGGTTAGCTCCTTCGGTCCTCCGATCGTTGTCAGAAGTAAGTTGGCCGCAGTGTTATCACTCATGGTTATGGCAGCACTGCATAATTCTCTTACTGTCATGCCATCCGTAAGATGCTTTTCTGTGACTGGTGAGTACTCAACCAAGTCATTCTGAGAATAGTGTATGCGGCGACCGAGTTGCTCTTGCCCGGCGTCAATACGGGATAATACCGCGCCACATAGCAGAACTTTAAAAGTGCTCATCATTGGAAAACGTTCTTCGGGGCGAAAACTCTCAAGGATCTTACCGCTGTTGAGATCCAGTTCGATGTAACCCACTCGTGCACCCAACTGATCTTCAGCATCTTTTACTTTCACCAGCGTTTCTGGGTGAGCAAAAACAGGAAGGCAAAATGCCGCAAAAAAGGGAATAAGGGCGACACGGAAATGTTGAATACTCATACTCTTCCTTTTTCAATATTATTGAAGCATTTATCAGGGTTATTGTCTCATGAGCGGATACATATTTGAATGTATTTAGAAAAATAAACAAATAGGGGTTCCGCGCACATTTCCCCGAAAAGTGCCACCTGACGTC

**mCherry-*Tmc1*-wt:**

GACGGATCGGGAGATCTCCCGATCCCCTATGGTGCACTCTCAGTACAATCTGCTCTGATGCCGCATAGTTAAGCCAGTATCTGCTCCCTGCTTGTGTGTTGGAGGTCGCTGAGTAGTGCGCGAGCAAAATTTAAGCTACAACAAGGCAAGGCTTGACCGACAATTGCATGAAGAATCTGCTTAGGGTTAGGCGTTTTGCGCTGCTTCGCGATGTACGGGCCAGATATACGCGTTGACATTGATTATTGACTAGTTATTAATAGTAATCAATTACGGGGTCATTAGTTCATAGCCCATATATGGAGTTCCGCGTTACATAACTTACGGTAAATGGCCCGCCTGGCTGACCGCCCAACGACCCCCGCCCATTGACGTCAATAATGACGTATGTTCCCATAGTAACGCCAATAGGGACTTTCCATTGACGTCAATGGGTGGAGTATTTACGGTAAACTGCCCACTTGGCAGTACATCAAGTGTATCATATGCCAAGTACGCCCCCTATTGACGTCAATGACGGTAAATGGCCCGCCTGGCATTATGCCCAGTACATGACCTTATGGGACTTTCCTACTTGGCAGTACATCTACGTATTAGTCATCGCTATTACCATGGTGATGCGGTTTTGGCAGTACATCAATGGGCGTGGATAGCGGTTTGACTCACGGGGATTTCCAAGTCTCCACCCCATTGACGTCAATGGGAGTTTGTTTTGGCACCAAAATCAACGGGACTTTCCAAAATGTCGTAACAACTCCGCCCCATTGACGCAAATGGGCGGTAGGCGTGTACGGTGGGAGGTCTATATAAGCAGAGCTCTCTGGCTAACTAGAGAACCCACTGCTTACTGGCTTATCGAAATTAATACGACTCACTATAGGGAGACCCAAGCTGGCTAGCGTTTAAACGGGCCCTCTAGACTCGAG**ATG**GTGAGCAAGGGCGAGGAGGATAACATGGCCATCATCAAGGAGTTCATGCGCTTCAAGGTGCACATGGAGGGCTCCGTGAACGGCCACGAGTTCGAGATCGAGGGCGAGGGCGAGGGCCGCCCCTACGAGGGCACCCAGACCGCCAAGCTGAAGGTGACCAAGGGTGGCCCCCTGCCCTTCGCCTGGGACATCCTGTCCCCTCAGTTCATGTACGGCTCCAAGGCCTACGTGAAGCACCCCGCCGACATCCCCGACTACTTGAAGCTGTCCTTCCCCGAGGGCTTCAAGTGGGAGCGCGTGATGAACTTCGAGGACGGCGGCGTGGTGACCGTGACCCAGGACTCCTCCCTGCAGGACGGCGAGTTCATCTACAAGGTGAAGCTGCGCGGCACCAACTTCCCCTCCGACGGCCCCGTAATGCAGAAGAAGACCATGGGCTGGGAGGCCTCCTCCGAGCGGATGTACCCCGAGGACGGCGCCCTGAAGGGCGAGATCAAGCAGAGGCTGAAGCTGAAGGACGGCGGCCACTACGACGCTGAGGTCAAGACCACCTACAAGGCCAAGAAGCCCGTGCAGCTGCCCGGCGCCTACAACGTCAACATCAAGTTGGACATCACCTCCCACAACGAGGACTACACCATCGTGGAACAGTACGAACGCGCCGAGGGCCGCCACTCCACCGGCGGCATGGACGAGCTGTACAAGAGTGGATACCTCATCTTTTGGGCTGTGAAGCGATCCCAGGAGTTCGCCCAGCAAGATCCTGACACCCTTGGGTGGTGGGAAAAAAATGAAATGAACATGGTAATGTCCCTCCTGGGGATGTTCTGTCCCACCCTGTTTGACTTATTTGCTGAACTGGAAGATTACCATCCTCTCATTGCTCTGAAGTGGCTCCTGGGGCGCATTTTTGCTCTTCTTCTAGGCAACTTGTATGTATTCATTCTCGCCTTGATGGATGAGATTAACAACAAGATTGAAGAGGAGAAGCTTGTGAAGGCCAATAAGCTTAAGTTTAAACCGCTGATCAGCCTCGACTGTGCCTTCTAGTTGCCAGCCATCTGTTGTTTGCCCCTCCCCCGTGCCTTCCTTGACCCTGGAAGGTGCCACTCCCACTGTCCTTTCCTAATAAAATGAGGAAATTGCATCGCATTGTCTGAGTAGGTGTCATTCTATTCTGGGGGGTGGGGTGGGGCAGGACAGCAAGGGGGAGGATTGGGAAGACAATAGCAGGCATGCTGGGGATGCGGTGGGCTCTATGGCTTCTGAGGCGGAAAGAACCAGCTGGGGCTCTAGGGGGTATCCCCACGCGCCCTGTAGCGGCGCATTAAGCGCGGCGGGTGTGGTGGTTACGCGCAGCGTGACCGCTACACTTGCCAGCGCCCTAGCGCCCGCTCCTTTCGCTTTCTTCCCTTCCTTTCTCGCCACGTTCGCCGGCTTTCCCCGTCAAGCTCTAAATCGGGGGCTCCCTTTAGGGTTCCGATTTAGTGCTTTACGGCACCTCGACCCCAAAAAACTTGATTAGGGTGATGGTTCACGTAGTGGGCCATCGCCCTGATAGACGGTTTTTCGCCCTTTGACGTTGGAGTCCACGTTCTTTAATAGTGGACTCTTGTTCCAAACTGGAACAACACTCAACCCTATCTCGGTCTATTCTTTTGATTTATAAGGGATTTTGCCGATTTCGGCCTATTGGTTAAAAAATGAGCTGATTTAACAAAAATTTAACGCGAATTAATTCTGTGGAATGTGTGTCAGTTAGGGTGTGGAAAGTCCCCAGGCTCCCCAGCAGGCAGAAGTATGCAAAGCATGCATCTCAATTAGTCAGCAACCAGGTGTGGAAAGTCCCCAGGCTCCCCAGCAGGCAGAAGTATGCAAAGCATGCATCTCAATTAGTCAGCAACCATAGTCCCGCCCCTAACTCCGCCCATCCCGCCCCTAACTCCGCCCAGTTCCGCCCATTCTCCGCCCCATGGCTGACTAATTTTTTTTATTTATGCAGAGGCCGAGGCCGCCTCTGCCTCTGAGCTATTCCAGAAGTAGTGAGGAGGCTTTTTTGGAGGCCTAGGCTTTTGCAAAAAGCTCCCGGGAGCTTGTATATCCATTTTCGGATCTGATCAAGAGACAGGATGAGGATCGTTTCGCATGATTGAACAAGATGGATTGCACGCAGGTTCTCCGGCCGCTTGGGTGGAGAGGCTATTCGGCTATGACTGGGCACAACAGACAATCGGCTGCTCTGATGCCGCCGTGTTCCGGCTGTCAGCGCAGGGGCGCCCGGTTCTTTTTGTCAAGACCGACCTGTCCGGTGCCCTGAATGAACTGCAGGACGAGGCAGCGCGGCTATCGTGGCTGGCCACGACGGGCGTTCCTTGCGCAGCTGTGCTCGACGTTGTCACTGAAGCGGGAAGGGACTGGCTGCTATTGGGCGAAGTGCCGGGGCAGGATCTCCTGTCATCTCACCTTGCTCCTGCCGAGAAAGTATCCATCATGGCTGATGCAATGCGGCGGCTGCATACGCTTGATCCGGCTACCTGCCCATTCGACCACCAAGCGAAACATCGCATCGAGCGAGCACGTACTCGGATGGAAGCCGGTCTTGTCGATCAGGATGATCTGGACGAAGAGCATCAGGGGCTCGCGCCAGCCGAACTGTTCGCCAGGCTCAAGGCGCGCATGCCCGACGGCGAGGATCTCGTCGTGACCCATGGCGATGCCTGCTTGCCGAATATCATGGTGGAAAATGGCCGCTTTTCTGGATTCATCGACTGTGGCCGGCTGGGTGTGGCGGACCGCTATCAGGACATAGCGTTGGCTACCCGTGATATTGCTGAAGAGCTTGGCGGCGAATGGGCTGACCGCTTCCTCGTGCTTTACGGTATCGCCGCTCCCGATTCGCAGCGCATCGCCTTCTATCGCCTTCTTGACGAGTTCTTCTGAGCGGGACTCTGGGGTTCGAAATGACCGACCAAGCGACGCCCAACCTGCCATCACGAGATTTCGATTCCACCGCCGCCTTCTATGAAAGGTTGGGCTTCGGAATCGTTTTCCGGGACGCCGGCTGGATGATCCTCCAGCGCGGGGATCTCATGCTGGAGTTCTTCGCCCACCCCAACTTGTTTATTGCAGCTTATAATGGTTACAAATAAAGCAATAGCATCACAAATTTCACAAATAAAGCATTTTTTTCACTGCATTCTAGTTGTGGTTTGTCCAAACTCATCAATGTATCTTATCATGTCTGTATACCGTCGACCTCTAGCTAGAGCTTGGCGTAATCATGGTCATAGCTGTTTCCTGTGTGAAATTGTTATCCGCTCACAATTCCACACAACATACGAGCCGGAAGCATAAAGTGTAAAGCCTGGGGTGCCTAATGAGTGAGCTAACTCACATTAATTGCGTTGCGCTCACTGCCCGCTTTCCAGTCGGGAAACCTGTCGTGCCAGCTGCATTAATGAATCGGCCAACGCGCGGGGAGAGGCGGTTTGCGTATTGGGCGCTCTTCCGCTTCCTCGCTCACTGACTCGCTGCGCTCGGTCGTTCGGCTGCGGCGAGCGGTATCAGCTCACTCAAAGGCGGTAATACGGTTATCCACAGAATCAGGGGATAACGCAGGAAAGAACATGTGAGCAAAAGGCCAGCAAAAGGCCAGGAACCGTAAAAAGGCCGCGTTGCTGGCGTTTTTCCATAGGCTCCGCCCCCCTGACGAGCATCACAAAAATCGACGCTCAAGTCAGAGGTGGCGAAACCCGACAGGACTATAAAGATACCAGGCGTTTCCCCCTGGAAGCTCCCTCGTGCGCTCTCCTGTTCCGACCCTGCCGCTTACCGGATACCTGTCCGCCTTTCTCCCTTCGGGAAGCGTGGCGCTTTCTCATAGCTCACGCTGTAGGTATCTCAGTTCGGTGTAGGTCGTTCGCTCCAAGCTGGGCTGTGTGCACGAACCCCCCGTTCAGCCCGACCGCTGCGCCTTATCCGGTAACTATCGTCTTGAGTCCAACCCGGTAAGACACGACTTATCGCCACTGGCAGCAGCCACTGGTAACAGGATTAGCAGAGCGAGGTATGTAGGCGGTGCTACAGAGTTCTTGAAGTGGTGGCCTAACTACGGCTACACTAGAAGAACAGTATTTGGTATCTGCGCTCTGCTGAAGCCAGTTACCTTCGGAAAAAGAGTTGGTAGCTCTTGATCCGGCAAACAAACCACCGCTGGTAGCGGTTTTTTTGTTTGCAAGCAGCAGATTACGCGCAGAAAAAAAGGATCTCAAGAAGATCCTTTGATCTTTTCTACGGGGTCTGACGCTCAGTGGAACGAAAACTCACGTTAAGGGATTTTGGTCATGAGATTATCAAAAAGGATCTTCACCTAGATCCTTTTAAATTAAAAATGAAGTTTTAAATCAATCTAAAGTATATATGAGTAAACTTGGTCTGACAGTTACCAATGCTTAATCAGTGAGGCACCTATCTCAGCGATCTGTCTATTTCGTTCATCCATAGTTGCCTGACTCCCCGTCGTGTAGATAACTACGATACGGGAGGGCTTACCATCTGGCCCCAGTGCTGCAATGATACCGCGAGACCCACGCTCACCGGCTCCAGATTTATCAGCAATAAACCAGCCAGCCGGAAGGGCCGAGCGCAGAAGTGGTCCTGCAACTTTATCCGCCTCCATCCAGTCTATTAATTGTTGCCGGGAAGCTAGAGTAAGTAGTTCGCCAGTTAATAGTTTGCGCAACGTTGTTGCCATTGCTACAGGCATCGTGGTGTCACGCTCGTCGTTTGGTATGGCTTCATTCAGCTCCGGTTCCCAACGATCAAGGCGAGTTACATGATCCCCCATGTTGTGCAAAAAAGCGGTTAGCTCCTTCGGTCCTCCGATCGTTGTCAGAAGTAAGTTGGCCGCAGTGTTATCACTCATGGTTATGGCAGCACTGCATAATTCTCTTACTGTCATGCCATCCGTAAGATGCTTTTCTGTGACTGGTGAGTACTCAACCAAGTCATTCTGAGAATAGTGTATGCGGCGACCGAGTTGCTCTTGCCCGGCGTCAATACGGGATAATACCGCGCCACATAGCAGAACTTTAAAAGTGCTCATCATTGGAAAACGTTCTTCGGGGCGAAAACTCTCAAGGATCTTACCGCTGTTGAGATCCAGTTCGATGTAACCCACTCGTGCACCCAACTGATCTTCAGCATCTTTTACTTTCACCAGCGTTTCTGGGTGAGCAAAAACAGGAAGGCAAAATGCCGCAAAAAAGGGAATAAGGGCGACACGGAAATGTTGAATACTCATACTCTTCCTTTTTCAATATTATTGAAGCATTTATCAGGGTTATTGTCTCATGAGCGGATACATATTTGAATGTATTTAGAAAAATAAACAAATAGGGGTTCCGCGCACATTTCCCCGAAAAGTGCCACCTGACGTC

(2) RNA editors.

CasRx and PspCas13b are highlighted in green, respectively.

**CasRx:**

GAACGCGCGGCGCACCGGGAAGCCCTCGCCCTCGAAACCGCTGGGCGCGGTGGTCACGGTGAGCACGGGACGTGCGACGGCGTCGGCGGGTGCGGATACGCGGGGCAGCGTCAGCGGGTTCTCGACGGTCACGGCGGGCATGCGGCCGCATAACTTACGGTAAATGGCCCGCCTGGCTGACCGCCCAACGACCCCCGCCCATTGACGTCAATAATGACGTATGTTCCCATAGTAACGCCAATAGGGACTTTCCATTGACGTCAATGGGTGGAGTATTTACGGTAAACTGCCCACTTGGCAGTACATCAAGTGTATCATATGCCAAGTACGCCCCCTATTGACGTCAATGACGGTAAATGGCCCGCCTGGCATTATGCCCAGTACATGACCTTATGGGACTTTCCTACTTGGCAGTACATCTACGTATTAGTCATCGCTATTACCATGGTCGAGGTGAGCCCCACGTTCTGCTTCACTCTCCCCATCTCCCCCCCCTCCCCACCCCCAATTTTGTATTTATTTATTTTTTAATTATTTTGTGCAGCGATGGGGGCGGGGGGGGGGGGGGGGCGCGCGCCAGGCGGGGCGGGGCGGGGCGAGGGGCGGGGCGGGGCGAGGCGGAGAGGTGCGGCGGCAGCCAATCAGAGCGGCGCGCTCCGAAAGTTTCCTTTTATGGCGAGGCGGCGGCGGCGGCGGCCCTATAAAAAGCGAAGCGCGCGGCGGGCGGGGAGTCGCTGCGACGCTGCCTTCGCCCCGTGCCCCGCTCCGCCGCCGCCTCGCGCCGCCCGCCCCGGCTCTGACTGACCGCGTTACTCCCACAGGTGAGCGGGCGGGACGGCCCTTCTCCTCCGGGCTGTAATTAGCGCTTGGTTTAATGACGGCTTGTTTCTTTTCTGTGGCTGCGTGAAAGCCTTGAGGGGCTCCGGGAGGGCCCTTTGTGCGGGGGGAGCGGCTCGGGGGGTGCGTGCGTGTGTGTGTGCGTGGGGAGCGCCGCGTGCGGCTCCGCGCTGCCCGGCGGCTGTGAGCGCTGCGGGCGCGGCGCGGGGCTTTGTGCGCTCCGCAGTGTGCGCGAGGGGAGCGCGGCCGGGGGCGGTGCCCCGCGGTGCGGGGGGGGCTGCGAGGGGAACAAAGGCTGCGTGCGGGGTGTGTGCGTGGGGGGGTGAGCAGGGGGTGTGGGCGCGTCGGTCGGGCTGCAACCCCCCCTGCACCCCCCTCCCCGAGTTGCTGAGCACGGCCCGGCTTCGGGTGCGGGGCTCCGTACGGGGCGTGGCGCGGGGCTCGCCGTGCCGGGCGGGGGGTGGCGGCAGGTGGGGGTGCCGGGCGGGGCGGGGCCGCCTCGGGCCGGGGAGGGCTCGGGGGAGGGGCGCGGCGGCCCCCGGAGCGCCGGCGGCTGTCGAGGCGCGGCGAGCCGCAGCCATTGCCTTTTATGGTAATCGTGCGAGAGGGCGCAGGGACTTCCTTTGTCCCAAATCTGTGCGGAGCCGAAATCTGGGAGGCGCCGCCGCACCCCCTCTAGCGGGCGCGGGGCGAAGCGGTGCGGCGCCGGCAGGAAGGAAATGGGCGGGGAGGGCCTTCGTGCGTCGCCGCGCCGCCGTCCCCTTCTCCCTCTCCAGCCTCGGGGCTGTCCGCGGGGGGACGGCTGCCTTCGGGGGGGACGGGGCAGGGCGGGGTTCGGCTTCTGGCGTGTGACCGGCGGCTCTAGAGCCTCTGCTAACCATGTTCATGCCTTCTTCTTTTTCCTACAGCTCCTGGGCAACGTGCTGGTTATTGTGCTGTCTCATCATTTTGGCAAAGAATTGGAATTCGCCGCCACCATGCCTAAAAAGAAAAGAAAGGTGGGTTCTGGTATGGAGAAGAAGAAGAGCTTCGCCAAGGGCATGGGAGTGAAGAGCACCCTGGTGTCCGGCTCTAAGGTGTACATGACCACATTTGCTGAGGGAAGCGACGCCAGGCTGGAGAAGATCGTGGAGGGCGATAGCATCAGATCCGTGAACGAGGGAGAGGCTTTCAGCGCCGAGATGGCTGACAAGAACGCTGGCTACAAGATCGGAAACGCCAAGTTTTCCCACCCAAAGGGCTACGCCGTGGTGGCTAACAACCCACTGTACACCGGACCAGTGCAGCAGGACATGCTGGGACTGAAGGAGACACTGGAGAAGAGGTACTTCGGCGAGTCCGCCGACGGAAACGATAACATCTGCATCCAGGTCATCCACAACATCCTGGATATCGAGAAGATCCTGGCTGAGTACATCACAAACGCCGCTTACGCCGTGAACAACATCTCCGGCCTGGACAAGGATATCATCGGCTTCGGAAAGTTTTCTACCGTGTACACATACGACGAGTTCAAGGATCCAGAGCACCACCGGGCCGCTTTTAACAACAACGACAAGCTGATCAACGCCATCAAGGCTCAGTACGACGAGTTCGATAACTTTCTGGATAACCCCAGGCTGGGCTACTTCGGACAGGCTTTCTTTTCTAAGGAGGGCAGAAACTACATCATCAACTACGGAAACGAGTGTTACGACATCCTGGCCCTGCTGAGCGGACTGAGGCACTGGGTGGTGCACAACAACGAGGAGGAGTCTCGGATCAGCCGCACCTGGCTGTACAACCTGGACAAGAACCTGGATAACGAGTACATCTCCACACTGAACTACCTGTACGACAGGATCACCAACGAGCTGACAAACAGCTTCTCCAAGAACTCTGCCGCTAACGTGAACTACATCGCTGAGACCCTGGGCATCAACCCAGCTGAGTTCGCTGAGCAGTACTTCAGATTTTCCATCATGAAGGAGCAGAAGAACCTGGGCTTCAACATCACAAAGCTGAGAGAAGTGATGCTGGACAGAAAGGATATGTCCGAGATCAGGAAGAACCACAAGGTGTTCGATTCTATCAGAACCAAGGTGTACACAATGATGGACTTTGTGATCTACAGGTACTACATCGAGGAGGATGCCAAGGTGGCCGCTGCCAACAAGAGCCTGCCCGACAACGAGAAGTCTCTGAGCGAGAAGGATATCTTCGTGATCAACCTGAGAGGCTCCTTTAACGACGATCAGAAGGACGCTCTGTACTACGATGAGGCCAACAGGATCTGGAGAAAGCTGGAGAACATCATGCACAACATCAAGGAGTTCCGGGGAAACAAGACCCGCGAGTACAAGAAGAAGGACGCTCCAAGGCTGCCTAGGATCCTGCCTGCTGGAAGGGACGTGAGCGCCTTCAGCAAGCTGATGTACGCCCTGACAATGTTTCTGGACGGAAAGGAGATCAACGATCTGCTGACCACACTGATCAACAAGTTCGACAACATCCAGTCTTTTCTGAAAGTGATGCCTCTGATCGGCGTGAACGCTAAGTTCGTGGAGGAGTACGCCTTCTTTAAGGACAGCGCCAAGATCGCTGATGAGCTGCGGCTGATCAAGTCCTTTGCCAGGATGGGAGAGCCAATCGCTGACGCTAGGAGAGCTATGTACATCGATGCCATCCGGATCCTGGGAACCAACCTGTCTTACGACGAGCTGAAGGCTCTGGCCGACACCTTCAGCCTGGATGAGAACGGCAACAAGCTGAAGAAGGGCAAGCACGGAATGCGCAACTTCATCATCAACAACGTGATCAGCAACAAGCGGTTTCACTACCTGATCAGATACGGCGACCCAGCTCACCTGCACGAGATCGCTAAGAACGAGGCCGTGGTGAAGTTCGTGCTGGGACGGATCGCCGATATCCAGAAGAAGCAGGGCCAGAACGGAAAGAACCAGATCGACCGCTACTACGAGACCTGCATCGGCAAGGATAAGGGAAAGTCCGTGTCTGAGAAGGTGGACGCTCTGACCAAGATCATCACAGGCATGAACTACGACCAGTTCGATAAGAAGAGATCTGTGATCGAGGACACCGGAAGGGAGAACGCCGAGAGAGAGAAGTTTAAGAAGATCATCAGCCTGTACCTGACAGTGATCTACCACATCCTGAAGAACATCGTGAACATCAACGCTAGATACGTGATCGGCTTCCACTGCGTGGAGCGCGATGCCCAGCTGTACAAGGAGAAGGGATACGACATCAACCTGAAGAAGCTGGAGGAGAAGGGCTTTAGCTCCGTGACCAAGCTGTGCGCTGGAATCGACGAGACAGCCCCCGACAAGAGGAAGGATGTGGAGAAGGAGATGGCCGAGAGAGCTAAGGAGAGCATCGACTCCCTGGAGTCTGCTAACCCTAAGCTGTACGCCAACTACATCAAGTACTCCGATGAGAAGAAGGCCGAGGAGTTCACCAGGCAGATCAACAGAGAGAAGGCCAAGACCGCTCTGAACGCCTACCTGAGGAACACAAAGTGGAACGTGATCATCCGGGAGGACCTGCTGCGCATCGATAACAAGACCTGTACACTGTTCCGGAACAAGGCTGTGCACCTGGAGGTGGCTCGCTACGTGCACGCCTACATCAACGACATCGCCGAGGTGAACTCCTACTTTCAGCTGTACCACTACATCATGCAGAGGATCATCATGAACGAGAGATACGAGAAGTCTAGCGGCAAGGTGTCTGAGTACTTCGACGCCGTGAACGATGAGAAGAAGTACAACGATAGACTGCTGAAGCTGCTGTGCGTGCCTTTCGGATACTGTATCCCACGGTTTAAGAACCTGAGCATCGAGGCCCTGTTCGACCGCAACGAGGCTGCCAAGTTTGATAAGGAGAAGAAGAAGGTGAGCGGCAACTCCGGTTCTGGTCTCGAGCCCAAGAAGAAGAGGAAAGTCCTCGAGGCTACTAACTTCAGCCTGCTGAAGCAGGCTGGAGACGTGGAGGAGAACCCTGGACCTATGCATATGGTGAGCAAGGGCGAGGAGCTGTTCACCGGGGTGGTGCCCATCCTGGTCGAGCTGGACGGCGACGTAAACGGCCACAAGTTCAGCGTGTCCGGCGAGGGCGAGGGCGATGCCACCTACGGCAAGCTGACCCTGAAGTTCATCTGCACCACCGGCAAGCTGCCCGTGCCCTGGCCCACCCTCGTGACCACCCTGACCTACGGCGTGCAGTGCTTCAGCCGCTACCCCGACCACATGAAGCAGCACGACTTCTTCAAGTCCGCCATGCCCGAAGGCTACGTCCAGGAGCGCACCATCTTCTTCAAGGACGACGGCAACTACAAGACCCGCGCCGAGGTGAAGTTCGAGGGCGACACCCTGGTGAACCGCATCGAGCTGAAGGGCATCGACTTCAAGGAGGACGGCAACATCCTGGGGCACAAGCTGGAGTACAACTACAACAGCCACAACGTCTATATCATGGCCGACAAGCAGAAGAACGGCATCAAGGTGAACTTCAAGATCCGCCACAACATCGAGGACGGCAGCGTGCAGCTCGCCGACCACTACCAGCAGAACACCCCCATCGGCGACGGCCCCGTGCTGCTGCCCGACAACCACTACCTGAGCACCCAGTCCGCCCTGAGCAAAGACCCCAACGAGAAGCGCGATCACATGGTCCTGCTGGAGTTCGTGACCGCCGCCGGGATCACTCTCGGCATGGACGAGCTGTACAAGTAAACGCTAGCTAGAATCAACCTCTGGATTACAAAATTTGTGAAAGATTGACTGGTATTCTTAACTATGTTGCTCCTTTTACGCTATGTGGATACGCTGCTTTAATGCCTTTGTATCATGCTATTGCTTCCCGTATGGCTTTCATTTTCTCCTCCTTGTATAAATCCTGGTTGCTGTCTCTTTATGAGGAGTTGTGGCCCGTTGTCAGGCAACGTGGCGTGGTGTGCACTGTGTTTGCTGACGCAACCCCCACTGGTTGGGGCATTGCCACCACCTGTCAGCTCCTTTCCGGGACTTTCGCTTTCCCCCTCCCTATTGCCACGGCGGAACTCATCGCCGCCTGCCTTGCCCGCTGCTGGACAGGGGCTCGGCTGTTGGGCACTGACAATTCCGTGGTGTTGTCGGGGAAATCATCGTCCTTTCCTTGGCTGCTCGCCTGTGTTGCCACCTGGATTCTGCGCGGGACGTCCTTCTGCTACGTCCCTTCGGCCCTCAATCCAGCGGACCTTCCTTCCCGCGGCCTGCTGCCGGCTCTGCGGCCTCTTCCGCGTCTTCGCCTTCGCCCTCAGACGAGTCGGATCTCCCTTTGGGCCGCCTCCCCGCATCGATACCGTCGACCTCGACTGTGCCTTCTAGTTGCCAGCCATCTGTTGTTTGCCCCTCCCCCGTGCCTTCCTTGACCCTGGAAGGTGCCACTCCCACTGTCCTTTCCTAATAAAATGAGGAAATTGCATCGCATTGTCTGAGTAGGTGTCATTCTATTCTGGGGGGTGGGGTGGGGCAGGACAGCAAGGGGGAGGATTGGGAAGACAATAGCAGGCATGCTGGGGATAACTTTAAATAATTGGCATTATTTAAAGTTAACGCGTACAAGTTTGTACAAAAAAGCTGAACGAGAAACGTAAAATGATATAAATATCAATATATTAAATTAGATTTTGCATAAAAAACAGACTACATAATACTGTAAAACACAACATATCCAGTCACTATGCTGCATTAATGAATCGGCCAACGCGCGGGGAGAGGCGGTTTGCGTATTGGGCGCTCTTCCGCTTCCTCGCTCACTGACTCGCTGCGCTCGGTCGTTCGGCTGCGGCGAGCGGTATCAGCTCACTCAAAGGCGGTAATACGGTTATCCACAGAATCAGGGGATAACGCAGGAAAGAACATGTGAGCAAAAGGCCAGCAAAAGGCCAGGAACCGTAAAAAGGCCGCGTTGCTGGCGTTTTTCCATAGGCTCCGCCCCCCTGACGAGCATCACAAAAATCGACGCTCAAGTCAGAGGTGGCGAAACCCGACAGGACTATAAAGATACCAGGCGTTTCCCCCTGGAAGCTCCCTCGTGCGCTCTCCTGTTCCGACCCTGCCGCTTACCGGATACCTGTCCGCCTTTCTCCCTTCGGGAAGCGTGGCGCTTTCTCATAGCTCACGCTGTAGGTATCTCAGTTCGGTGTAGGTCGTTCGCTCCAAGCTGGGCTGTGTGCACGAACCCCCCGTTCAGCCCGACCGCTGCGCCTTATCCGGTAACTATCGTCTTGAGTCCAACCCGGTAAGACACGACTTATCGCCACTGGCAGCAGCCACTGGTAACAGGATTAGCAGAGCGAGGTATGTAGGCGGTGCTACAGAGTTCTTGAAGTGGTGGCCTAACTACGGCTACACTAGAAGAACAGTATTTGGTATCTGCGCTCTGCTGAAGCCAGTTACCTTCGGAAAAAGAGTTGGTAGCTCTTGATCCGGCAAACAAACCACCGCTGGTAGCGGTGGTTTTTTTGTTTGCAAGCAGCAGATTACGCGCAGAAAAAAAGGATCTCAAGAAGATCCTTTGATCTTTTCTACGGGGTCTGACGCTCAGTGGAACGAAAACTCACGTTAAGGGATTTTGGTCATGAGATTATCAAAAAGGATCTTCACCTAGATCCTTTTAAATTAAAAATGAAGTTTTAAATCAATCTAAAGTATATATGAGTAAACTTGGTCTGACAGTTACCAATGCTTAATCAGTGAGGCACCTATCTCAGCGATCTGTCTATTTCGTTCATCCATAGTTGCCTGACTCCCCGTCGTGTAGATAACTACGATACGGGAGGGCTTACCATCTGGCCCCAGTGCTGCAATGATACCGCGAGACCCACGCTCACCGGCTCCAGATTTATCAGCAATAAACCAGCCAGCCGGAAGGGCCGAGCGCAGAAGTGGTCCTGCAACTTTATCCGCCTCCATCCAGTCTATTAATTGTTGCCGGGAAGCTAGAGTAAGTAGTTCGCCAGTTAATAGTTTGCGCAACGTTGTTGCCATTGCTACAGGCATCGTGGTGTCACGCTCGTCGTTTGGTATGGCTTCATTCAGCTCCGGTTCCCAACGATCAAGGCGAGTTACATGATCCCCCATGTTGTGCAAAAAAGCGGTTAGCTCCTTCGGTCCTCCGATCGTTGTCAGAAGTAAGTTGGCCGCAGTGTTATCACTCATGGTTATGGCAGCACTGCATAATTCTCTTACTGTCATGCCATCCGTAAGATGCTTTTCTGTGACTGGTGAGTACTCAACCAAGTCATTCTGAGAATAGTGTATGCGGCGACCGAGTTGCTCTTGCCCGGCGTCAATACGGGATAATACCGCGCCACATAGCAGAACTTTAAAAGTGCTCATCATTGGAAAACGTTCTTCGGGGCGAAAACTCTCAAGGATCTTACCGCTGTTGAGATCCAGTTCGATGTAACCCACTCGTGCACCCAACTGATCTTCAGCATCTTTTACTTTCACCAGCGTTTCTGGGTGAGCAAAAACAGGAAGGCAAAATGCCGCAAAAAAGGGAATAAGGGCGACACGGAAATGTTGAATACTCATACTCTTCCTTTTTCAATATTATTGAAGCATTTATCAGGGTTATTGTCTCATGAGCGGATACATATTTGAATGTATTTAGAAAAATAAACAAATAGGGGTTCCGCGCACATTTCCCCGAAAAGTGCCACCTGACGTCTAAGAAACCATTATTATCATGACATTAACCTATAAAAATCAAATAATGATTTTATTTTGACTGATAGTGACCTGTTCGTTGCAACAAATTGATGAGCAATGCTTTTTTATAATGCCAACTTTGTACAAAAAAGCAGGCTGTCGACGATGTAGGTCACGGTCTCGAAGCCGCGGTGCGGGTGCCAGGGCGTGCCCTTGGGCTCCCCGGGCGCGTACTCCACCTCACCCATCTGGTCCATCATGATGAACGGGTCGAGGTGGCGGTAGTTGATCCCGGC

**PspCas13b:**

GAACGCGCGGCGCACCGGGAAGCCCTCGCCCTCGAAACCGCTGGGCGCGGTGGTCACGGTGAGCACGGGACGTGCGACGGCGTCGGCGGGTGCGGATACGCGGGGCAGCGTCAGCGGGTTCTCGACGGTCACGGCGGGCATGCGGCCGCATAACTTACGGTAAATGGCCCGCCTGGCTGACCGCCCAACGACCCCCGCCCATTGACGTCAATAATGACGTATGTTCCCATAGTAACGCCAATAGGGACTTTCCATTGACGTCAATGGGTGGAGTATTTACGGTAAACTGCCCACTTGGCAGTACATCAAGTGTATCATATGCCAAGTACGCCCCCTATTGACGTCAATGACGGTAAATGGCCCGCCTGGCATTATGCCCAGTACATGACCTTATGGGACTTTCCTACTTGGCAGTACATCTACGTATTAGTCATCGCTATTACCATGGTCGAGGTGAGCCCCACGTTCTGCTTCACTCTCCCCATCTCCCCCCCCTCCCCACCCCCAATTTTGTATTTATTTATTTTTTAATTATTTTGTGCAGCGATGGGGGCGGGGGGGGGGGGGGGGCGCGCGCCAGGCGGGGCGGGGCGGGGCGAGGGGCGGGGCGGGGCGAGGCGGAGAGGTGCGGCGGCAGCCAATCAGAGCGGCGCGCTCCGAAAGTTTCCTTTTATGGCGAGGCGGCGGCGGCGGCGGCCCTATAAAAAGCGAAGCGCGCGGCGGGCGGGGAGTCGCTGCGACGCTGCCTTCGCCCCGTGCCCCGCTCCGCCGCCGCCTCGCGCCGCCCGCCCCGGCTCTGACTGACCGCGTTACTCCCACAGGTGAGCGGGCGGGACGGCCCTTCTCCTCCGGGCTGTAATTAGCGCTTGGTTTAATGACGGCTTGTTTCTTTTCTGTGGCTGCGTGAAAGCCTTGAGGGGCTCCGGGAGGGCCCTTTGTGCGGGGGGAGCGGCTCGGGGGGTGCGTGCGTGTGTGTGTGCGTGGGGAGCGCCGCGTGCGGCTCCGCGCTGCCCGGCGGCTGTGAGCGCTGCGGGCGCGGCGCGGGGCTTTGTGCGCTCCGCAGTGTGCGCGAGGGGAGCGCGGCCGGGGGCGGTGCCCCGCGGTGCGGGGGGGGCTGCGAGGGGAACAAAGGCTGCGTGCGGGGTGTGTGCGTGGGGGGGTGAGCAGGGGGTGTGGGCGCGTCGGTCGGGCTGCAACCCCCCCTGCACCCCCCTCCCCGAGTTGCTGAGCACGGCCCGGCTTCGGGTGCGGGGCTCCGTACGGGGCGTGGCGCGGGGCTCGCCGTGCCGGGCGGGGGGTGGCGGCAGGTGGGGGTGCCGGGCGGGGCGGGGCCGCCTCGGGCCGGGGAGGGCTCGGGGGAGGGGCGCGGCGGCCCCCGGAGCGCCGGCGGCTGTCGAGGCGCGGCGAGCCGCAGCCATTGCCTTTTATGGTAATCGTGCGAGAGGGCGCAGGGACTTCCTTTGTCCCAAATCTGTGCGGAGCCGAAATCTGGGAGGCGCCGCCGCACCCCCTCTAGCGGGCGCGGGGCGAAGCGGTGCGGCGCCGGCAGGAAGGAAATGGGCGGGGAGGGCCTTCGTGCGTCGCCGCGCCGCCGTCCCCTTCTCCCTCTCCAGCCTCGGGGCTGTCCGCGGGGGGACGGCTGCCTTCGGGGGGGACGGGGCAGGGCGGGGTTCGGCTTCTGGCGTGTGACCGGCGGCTCTAGAGCCTCTGCTAACCATGTTCATGCCTTCTTCTTTTTCCTACAGCTCCTGGGCAACGTGCTGGTTATTGTGCTGTCTCATCATTTTGGCAAAGAATTGGAATTCGCCGCCACCATGAACATCCCCGCTCTGGTGGAAAACCAGAAGAAGTACTTTGGCACCTACAGCGTGATGGCCATGCTGAACGCTCAGACCGTGCTGGACCACATCCAGAAGGTGGCCGATATTGAGGGCGAGCAGAACGAGAACAACGAGAATCTGTGGTTTCACCCCGTGATGAGCCACCTGTACAACGCCAAGAACGGCTACGACAAGCAGCCCGAGAAAACCATGTTCATCATCGAGCGGCTGCAGAGCTACTTCCCATTCCTGAAGATCATGGCCGAGAACCAGAGAGAGTACAGCAACGGCAAGTACAAGCAGAACCGCGTGGAAGTGAACAGCAACGACATCTTCGAGGTGCTGAAGCGCGCCTTCGGCGTGCTGAAGATGTACAGGGACCTGACCAACCACTACAAGACCTACGAGGAAAAGCTGAACGACGGCTGCGAGTTCCTGACCAGCACAGAGCAACCTCTGAGCGGCATGATCAACAACTACTACACAGTGGCCCTGCGGAACATGAACGAGAGATACGGCTACAAGACAGAGGACCTGGCCTTCATCCAGGACAAGCGGTTCAAGTTCGTGAAGGACGCCTACGGCAAGAAAAAGTCCCAAGTGAATACCGGATTCTTCCTGAGCCTGCAGGACTACAACGGCGACACACAGAAGAAGCTGCACCTGAGCGGAGTGGGAATCGCCCTGCTGATCTGCCTGTTCCTGGACAAGCAGTACATCAACATCTTTCTGAGCAGGCTGCCCATCTTCTCCAGCTACAATGCCCAGAGCGAGGAACGGCGGATCATCATCAGATCCTTCGGCATCAACAGCATCAAGCTGCCCAAGGACCGCATCCACAGCGAGAAGTCCAACAAGAGCGTGGCCATGGATATGCTCAACGAAGTGAAGCGGTGCCCCGACGAGCTGTTCACAACACTGTCTGCCGAGAAGCAGTCCCGGTTCAGAATCATCAGCGACGACCACAATGAAGTGCTGATGAAGCGGAGCAGCGACAGATTCGTGCCTCTGCTGCTGCAGTATATCGATTACGGCAAGCTGTTCGACCACATCAGGTTCCACGTGAACATGGGCAAGCTGAGATACCTGCTGAAGGCCGACAAGACCTGCATCGACGGCCAGACCAGAGTCAGAGTGATCGAGCAGCCCCTGAACGGCTTCGGCAGACTGGAAGAGGCCGAGACAATGCGGAAGCAAGAGAACGGCACCTTCGGCAACAGCGGCATCCGGATCAGAGACTTCGAGAACATGAAGCGGGACGACGCCAATCCTGCCAACTATCCCTACATCGTGGACACCTACACACACTACATCCTGGAAAACAACAAGGTCGAGATGTTTATCAACGACAAAGAGGACAGCGCCCCACTGCTGCCCGTGATCGAGGATGATAGATACGTGGTCAAGACAATCCCCAGCTGCCGGATGAGCACCCTGGAAATTCCAGCCATGGCCTTCCACATGTTTCTGTTCGGCAGCAAGAAAACCGAGAAGCTGATCGTGGACGTGCACAACCGGTACAAGAGACTGTTCCAGGCCATGCAGAAAGAAGAAGTGACCGCCGAGAATATCGCCAGCTTCGGAATCGCCGAGAGCGACCTGCCTCAGAAGATCCTGGATCTGATCAGCGGCAATGCCCACGGCAAGGATGTGGACGCCTTCATCAGACTGACCGTGGACGACATGCTGACCGACACCGAGCGGAGAATCAAGAGATTCAAGGACGACCGGAAGTCCATTCGGAGCGCCGACAACAAGATGGGAAAGAGAGGCTTCAAGCAGATCTCCACAGGCAAGCTGGCCGACTTCCTGGCCAAGGACATCGTGCTGTTTCAGCCCAGCGTGAACGATGGCGAGAACAAGATCACCGGCCTGAACTACCGGATCATGCAGAGCGCCATTGCCGTGTACGATAGCGGCGACGATTACGAGGCCAAGCAGCAGTTCAAGCTGATGTTCGAGAAGGCCCGGCTGATCGGCAAGGGCACAACAGAGCCTCATCCATTTCTGTACAAGGTGTTCGCCCGCAGCATCCCCGCCAATGCCGTCGAGTTCTACGAGCGCTACCTGATCGAGCGGAAGTTCTACCTGACCGGCCTGTCCAACGAGATCAAGAAAGGCAACAGAGTGGATGTGCCCTTCATCCGGCGGGACCAGAACAAGTGGAAAACACCCGCCATGAAGACCCTGGGCAGAATCTACAGCGAGGATCTGCCCGTGGAACTGCCCAGACAGATGTTCGACAATGAGATCAAGTCCCACCTGAAGTCCCTGCCACAGATGGAAGGCATCGACTTCAACAATGCCAACGTGACCTATCTGATCGCCGAGTACATGAAGAGAGTGCTGGACGACGACTTCCAGACCTTCTACCAGTGGAACCGCAACTACCGGTACATGGACATGCTTAAGGGCGAGTACGACAGAAAGGGCTCCCTGCAGCACTGCTTCACCAGCGTGGAAGAGAGAGAAGGCCTCTGGAAAGAGCGGGCCTCCAGAACAGAGCGGTACAGAAAGCAGGCCAGCAACAAGATCCGCAGCAACCGGCAGATGAGAAACGCCAGCAGCGAAGAGATCGAGACAATCCTGGATAAGCGGCTGAGCAACAGCCGGAACGAGTACCAGAAAAGCGAGAAAGTGATCCGGCGCTACAGAGTGCAGGATGCCCTGCTGTTTCTGCTGGCCAAAAAGACCCTGACCGAACTGGCCGATTTCGACGGCGAGAGGTTCAAACTGAAAGAAATCATGCCCGACGCCGAGAAGGGAATCCTGAGCGAGATCATGCCCATGAGCTTCACCTTCGAGAAAGGCGGCAAGAAGTACACCATCACCAGCGAGGGCATGAAGCTGAAGAACTACGGCGACTTCTTTGTGCTGGCTAGCGACAAGAGGATCGGCAACCTGCTGGAACTCGTGGGCAGCGACATCGTGTCCAAAGAGGATATCATGGAAGAGTTCAACAAATACGACCAGTGCAGGCCCGAGATCAGCTCCATCGTGTTCAACCTGGAAAAGTGGGCCTTCGACACATACCCCGAGCTGTCTGCCAGAGTGGACCGGGAAGAGAAGGTGGACTTCAAGAGCATCCTGAAAATCCTGCTGAACAACAAGAACATCAACAAAGAGCAGAGCGACATCCTGCGGAAGATCCGGAACGCCTTCGATCACAACAATTACCCCGACAAAGGCGTGGTGGAAATCAAGGCCCTGCCTGAGATCGCCATGAGCATCAAGAAGGCCTTTGGGGAGTACGCCATCATGAAGGCCGGTAGTGGGAGCAACGGCAGCAGCGGATCCCTGCCTCCACTTGAAAGACTGACACTGGGGTCCGGTAGATCCCTCGAGGCTACTAACTTCAGCCTGCTGAAGCAGGCTGGAGACGTGGAGGAGAACCCTGGACCTTAGCTAGAATCAACCTCTGGATTACAAAATTTGTGAAAGATTGACTGGTATTCTTAACTATGTTGCTCCTTTTACGCTATGTGGATACGCTGCTTTAATGCCTTTGTATCATGCTATTGCTTCCCGTATGGCTTTCATTTTCTCCTCCTTGTATAAATCCTGGTTGCTGTCTCTTTATGAGGAGTTGTGGCCCGTTGTCAGGCAACGTGGCGTGGTGTGCACTGTGTTTGCTGACGCAACCCCCACTGGTTGGGGCATTGCCACCACCTGTCAGCTCCTTTCCGGGACTTTCGCTTTCCCCCTCCCTATTGCCACGGCGGAACTCATCGCCGCCTGCCTTGCCCGCTGCTGGACAGGGGCTCGGCTGTTGGGCACTGACAATTCCGTGGTGTTGTCGGGGAAATCATCGTCCTTTCCTTGGCTGCTCGCCTGTGTTGCCACCTGGATTCTGCGCGGGACGTCCTTCTGCTACGTCCCTTCGGCCCTCAATCCAGCGGACCTTCCTTCCCGCGGCCTGCTGCCGGCTCTGCGGCCTCTTCCGCGTCTTCGCCTTCGCCCTCAGACGAGTCGGATCTCCCTTTGGGCCGCCTCCCCGCATCGATACCGTCGACCTCGACTGTGCCTTCTAGTTGCCAGCCATCTGTTGTTTGCCCCTCCCCCGTGCCTTCCTTGACCCTGGAAGGTGCCACTCCCACTGTCCTTTCCTAATAAAATGAGGAAATTGCATCGCATTGTCTGAGTAGGTGTCATTCTATTCTGGGGGGTGGGGTGGGGCAGGACAGCAAGGGGGAGGATTGGGAAGACAATAGCAGGCATGCTGGGGATAACTTTAAATAATTGGCATTATTTAAAGTTAACGCGTACAAGTTTGTACAAAAAAGCTGAACGAGAAACGTAAAATGATATAAATATCAATATATTAAATTAGATTTTGCATAAAAAACAGACTACATAATACTGTAAAACACAACATATCCAGTCACTATGCTGCATTAATGAATCGGCCAACGCGCGGGGAGAGGCGGTTTGCGTATTGGGCGCTCTTCCGCTTCCTCGCTCACTGACTCGCTGCGCTCGGTCGTTCGGCTGCGGCGAGCGGTATCAGCTCACTCAAAGGCGGTAATACGGTTATCCACAGAATCAGGGGATAACGCAGGAAAGAACATGTGAGCAAAAGGCCAGCAAAAGGCCAGGAACCGTAAAAAGGCCGCGTTGCTGGCGTTTTTCCATAGGCTCCGCCCCCCTGACGAGCATCACAAAAATCGACGCTCAAGTCAGAGGTGGCGAAACCCGACAGGACTATAAAGATACCAGGCGTTTCCCCCTGGAAGCTCCCTCGTGCGCTCTCCTGTTCCGACCCTGCCGCTTACCGGATACCTGTCCGCCTTTCTCCCTTCGGGAAGCGTGGCGCTTTCTCATAGCTCACGCTGTAGGTATCTCAGTTCGGTGTAGGTCGTTCGCTCCAAGCTGGGCTGTGTGCACGAACCCCCCGTTCAGCCCGACCGCTGCGCCTTATCCGGTAACTATCGTCTTGAGTCCAACCCGGTAAGACACGACTTATCGCCACTGGCAGCAGCCACTGGTAACAGGATTAGCAGAGCGAGGTATGTAGGCGGTGCTACAGAGTTCTTGAAGTGGTGGCCTAACTACGGCTACACTAGAAGAACAGTATTTGGTATCTGCGCTCTGCTGAAGCCAGTTACCTTCGGAAAAAGAGTTGGTAGCTCTTGATCCGGCAAACAAACCACCGCTGGTAGCGGTGGTTTTTTTGTTTGCAAGCAGCAGATTACGCGCAGAAAAAAAGGATCTCAAGAAGATCCTTTGATCTTTTCTACGGGGTCTGACGCTCAGTGGAACGAAAACTCACGTTAAGGGATTTTGGTCATGAGATTATCAAAAAGGATCTTCACCTAGATCCTTTTAAATTAAAAATGAAGTTTTAAATCAATCTAAAGTATATATGAGTAAACTTGGTCTGACAGTTACCAATGCTTAATCAGTGAGGCACCTATCTCAGCGATCTGTCTATTTCGTTCATCCATAGTTGCCTGACTCCCCGTCGTGTAGATAACTACGATACGGGAGGGCTTACCATCTGGCCCCAGTGCTGCAATGATACCGCGAGACCCACGCTCACCGGCTCCAGATTTATCAGCAATAAACCAGCCAGCCGGAAGGGCCGAGCGCAGAAGTGGTCCTGCAACTTTATCCGCCTCCATCCAGTCTATTAATTGTTGCCGGGAAGCTAGAGTAAGTAGTTCGCCAGTTAATAGTTTGCGCAACGTTGTTGCCATTGCTACAGGCATCGTGGTGTCACGCTCGTCGTTTGGTATGGCTTCATTCAGCTCCGGTTCCCAACGATCAAGGCGAGTTACATGATCCCCCATGTTGTGCAAAAAAGCGGTTAGCTCCTTCGGTCCTCCGATCGTTGTCAGAAGTAAGTTGGCCGCAGTGTTATCACTCATGGTTATGGCAGCACTGCATAATTCTCTTACTGTCATGCCATCCGTAAGATGCTTTTCTGTGACTGGTGAGTACTCAACCAAGTCATTCTGAGAATAGTGTATGCGGCGACCGAGTTGCTCTTGCCCGGCGTCAATACGGGATAATACCGCGCCACATAGCAGAACTTTAAAAGTGCTCATCATTGGAAAACGTTCTTCGGGGCGAAAACTCTCAAGGATCTTACCGCTGTTGAGATCCAGTTCGATGTAACCCACTCGTGCACCCAACTGATCTTCAGCATCTTTTACTTTCACCAGCGTTTCTGGGTGAGCAAAAACAGGAAGGCAAAATGCCGCAAAAAAGGGAATAAGGGCGACACGGAAATGTTGAATACTCATACTCTTCCTTTTTCAATATTATTGAAGCATTTATCAGGGTTATTGTCTCATGAGCGGATACATATTTGAATGTATTTAGAAAAATAAACAAATAGGGGTTCCGCGCACATTTCCCCGAAAAGTGCCACCTGACGTCTAAGAAACCATTATTATCATGACATTAACCTATAAAAATCAAATAATGATTTTATTTTGACTGATAGTGACCTGTTCGTTGCAACAAATTGATGAGCAATGCTTTTTTATAATGCCAACTTTGTACAAAAAAGCAGGCTGTCGACGATGTAGGTCACGGTCTCGAAGCCGCGGTGCGGGTGCCAGGGCGTGCCCTTGGGCTCCCCGGGCGCGTACTCCACCTCACCCATCTGGTCCATCATGATGAACGGGTCGAGGTGGCGGTAGTTGATCCCGGC

(3) sgRNA expression vectors.

Direct repeat (DR) of CasRx and PspCas13 are highlighted in red, respectively. The BspQI restriction enzyme cutting site of CasRx sgRNA expression vector and BbsI restriction enzyme cutting site of PspCas13b sgRNA expression vector are capitalized.

**CasRx-sgRNA expression vector:**

TGAGCAAAAGGCCAGCAAAAGGCCAGGAACCGTAAAAAGGCCGCGTTGCTGGCGTTTTTCCATAGGCTCCGCCCCCCTGACGAGCATCACAAAAATCGACGCTCAAGTCAGAGGTGGCGAAACCCGACAGGACTATAAAGATACCAGGCGTTTCCCCCTGGAAGCTCCCTCGTGCGCTCTCCTGTTCCGACCCTGCCGCTTACCGGATACCTGTCCGCCTTTCTCCCTTCGGGAAGCGTGGCGCTTTCTCAATGCTCACGCTGTAGGTATCTCAGTTCGGTGTAGGTCGTTCGCTCCAAGCTGGGCTGTGTGCACGAACCCCCCGTTCAGCCCGACCGCTGCGCCTTATCCGGTAACTATCGTCTTGAGTCCAACCCGGTAAGACACGACTTATCGCCACTGGCAGCAGCCACTGGTAACAGGATTAGCAGAGCGAGGTATGTAGGCGGTGCTACAGAGTTCTTGAAGTGGTGGCCTAACTACGGCTACACTAGAAGGACAGTATTTGGTATCTGCGCTCTGCTGAAGCCAGTTACCTTCGGAAAAAGAGTTGGTAGCTCTTGATCCGGCAAACAAACCACCGCTGGTAGCGGTGGTTTTTTTGTTTGCAAGCAGCAGATTACGCGCAGAAAAAAAGGATCTCAAGAAGATCCTTTGATCTTTTCTACGGGGTCTGACGCTCAGTGGAACGAAAACTCACGTTAAGGGATTTTGGTCATGAGATTATCAAAAAGGATCTTCACCTAGATCCTTTTAAATTAAAAATGAAGTTTTAAATCAATCTAAAGTATATATGAGTAAACTTGGTCTGACAGTTACCAATGCTTAATCAGTGAGGCACCTATCTCAGCGATCTGTCTATTTCGTTCATCCATAGTTGCCTGACTCCCCGTCGTGTAGATAACTACGATACGGGAGGGCTTACCATCTGGCCCCAGTGCTGCAATGATACCGCGAGATCCACGCTCACCGGCTCCAGATTTATCAGCAATAAACCAGCCAGCCGGAAGGGCCGAGCGCAGAAGTGGTCCTGCAACTTTATCCGCCTCCATCCAGTCTATTAATTGTTGCCGGGAAGCTAGAGTAAGTAGTTCGCCAGTTAATAGTTTGCGCAACGTTGTTGCCATTGCTACAGGCATCGTGGTGTCACGCTCGTCGTTTGGTATGGCTTCATTCAGCTCCGGTTCCCAACGATCAAGGCGAGTTACATGATCCCCCATGTTGTGCAAAAAAGCGGTTAGCTCCTTCGGTCCTCCGATCGTTGTCAGAAGTAAGTTGGCCGCAGTGTTATCACTCATGGTTATGGCAGCACTGCATAATTCTCTTACTGTCATGCCATCCGTAAGATGCTTTTCTGTGACTGGTGAGTACTCAACCAAGTCATTCTGAGAATAGTGTATGCGGCGACCGAGTTGCTCTTGCCCGGCGTCAATACGGGATAATACCGCGCCACATAGCAGAACTTTAAAAGTGCTCATCATTGGAAAACGTTCTTCGGGGCGAAAACTCTCAAGGATCTTACCGCTGTTGAGATCCAGTTCGATGTAACCCACTCGTGCACCCAACTGATCTTCAGCATCTTTTACTTTCACCAGCGTTTCTGGGTGAGCAAAAACAGGAAGGCAAAATGCCGCAAAAAAGGGAATAAGGGCGACACGGAAATGTTGAATACTCATACTCTTCCTTTTTCAATATTATTGAAGCATTTATCAGGGTTATTGTCTCATGAGCGGATACATATTTGAATGTATTTAGAAAAATAAACAAATAGGGGTTCCGCGCACATTTCCCCGAAAAGTGCCACCTGACGCGCCCTGTAGCGGCGCATTAAGCGCGGCGGGTGTGGTGGTTACGCGCAGCGTGACCGCTACACTTGCCAGCGCCCTAGCGCCCGCTCCTTTCGCTTTCTTCCCTTCCTTTCTCGCCACGTTCGCCGGCTTTCCCCGTCAAGCTCTAAATCGGGGGCTCCCTTTAGGGTTCCGATTTAGTGCTTTACGGCACCTCGACCCCAAAAAACTTGATTAGGGTGATGGTTCACGTAGTGGGCCATCGCCCTGATAGACGGTTTTTCGCCCTTTGACGTTGGAGTCCACGTTCTTTAATAGTGGACTCTTGTTCCAAACTGGAACAACACTCAACCCTATCTCGGTCTATTCTTTTGATTTATAAGGGATTTTGCCGATTTCGGCCTATTGGTTAAAAAATGAGCTGATTTAACAAAAATTTAACGCGAATTTTAACAAAATATTAACGCTTACAATTTGCCATTCGCCATTCAGGCTGCGCAACTGTTGGGAAGGGCGATCGGTGCGGGCCTCTTCGCTATTACGCCAGCCCAAGCTACCATGATAAGTAAGTAATATTAAGGTACGGGAGGTACTTGGAGCGGCCGCAATAAAATATCTTTATTTTCATTACATCTGTGTGTTGGTTTTTTGTGTGAATCGATAGTACTAACATACGCTCTCCATCAAAACAAAACGAAACAAAACAAACTAGCAAAATAGGCTGTCCCCAGTGCAAGTGCAGGTGCCAGAACATTTCTCTATCGATAGGTACCGATTAGTGAACGGATCTCGACGGTATCGATCACGAGACTAGCCTCGAGCGGCCGCCCCCTTCACCGAGGGCCTATTTCCCATGATTCCTTCATATTTGCATATACGATACAAGGCTGTTAGAGAGATAATTGGAATTAATTTGACTGTAAACACAAAGATATTAGTACAAAATACGTGACGTAGAAAGTAATAATTTCTTGGGTAGTTTGCAGTTTTAAAATTATGTTTTAAAATGGACTATCATATGCTTACCGTAACTTGAAAGTATTTCGATTTCTTGGCTTTATATATCTTGTGGAAAGGACGAAACACCGCAAGTAAACCCCTACCAACTGGTCGGGGTTTGA**AACAGAAGAGCCTCGAGGCTCTTCTCAA**GTAAACCCCTACCAACTGGTCGGGGTTTGAAACGAAGACTTTTTTTTTCGCTTCCTCGCTCACTGACTCGCTGCGCTCGGTCGTTCGGCTGCGGCGAGCGGTATCAGCTCACTCAAAGGCGGTAATACGGTCCTCGAGACAAATGGCAGTATTCATCCACAATTTTAAAAGAAAAGGGGGGATTGGGGGGTACAGTGCAGGGGAAAGAATAGTAGACATAATAGCAACAGACATACAAACTAAAGAATTACAAAAACAAATTACAAAAATTCAAAATTTTCGGGTTTATTACAGGGACAGCAGAGATCCACTTTGGCCGCGGCTCGAGGGGGTTGGGGTTGCGCCTTTTCCAAGGCAGCCCTGGGTTTGCGCAGGGACGCGGCTGCTCTGGGCGTGGTTCCGGGAAACGCAGCGGCGCCGACCCTGGGACTCGCACATTCTTCACGTCCGTTCGCAGCGTCACCCGGATCTTCGCCGCTACCCTTGTGGGCCCCCCGGCGACGCTTCCTGCTCCGCCCCTAAGTCGGGAAGGTTCCTTGCGGTTCGCGGCGTGCCGGACGTGACAAACGGAAGCCGCACGTCTCACTAGTACCCTCGCAGACGGACAGCGCCAGGGAGCAATGGCAGCGCGCCGACCGCGATGGGCTGTGGCCAATAGCGGCTGCTCAGCAGGGCGCGCCGAGAGCAGCGGCCGGGAAGGGGCGGTGCGGGAGGCGGGGTGTGGGGCGGTAGTGTGGGCCCTGTTCCTGCCCGCGCGGTGTTCCGCATTCTGCAAGCCTCCGGAGCGCACGTCGGCAGTCGGCTCCCTCGTTGACCGAATCACCGACCTCTCTCCCCAGGGGGATCCACCGGAGCTTACCATGGTGAGCAAGGGCGAGGAGCTGTTCACCGGGGTGGTGCCCATCCTGGTCGAGCTGGACGGCGACGTAAACGGCCACAAGTTCAGCGTGTCCGGCGAGGGCGAGGGCGATGCCACCTACGGCAAGCTGACCCTGAAGTTCATCTGCACCACCGGCAAGCTGCCCGTGCCCTGGCCCACCCTCGTGACCACCCTGACCTACGGCGTGCAGTGCTTCAGCCGCTACCCCGACCACATGAAGCAGCACGACTTCTTCAAGTCCGCCATGCCCGAAGGCTACGTCCAGGAGCGCACCATCTTCTTCAAGGACGACGGCAACTACAAGACCCGCGCCGAGGTGAAGTTCGAGGGCGACACCCTGGTGAACCGCATCGAGCTGAAGGGCATCGACTTCAAGGAGGACGGCAACATCCTGGGGCACAAGCTGGAGTACAACTACAACAGCCACAACGTCTATATCATGGCCGACAAGCAGAAGAACGGCATCAAGGTGAACTTCAAGATCCGCCACAACATCGAGGACGGCAGCGTGCAGCTCGCCGACCACTACCAGCAGAACACCCCCATCGGCGACGGCCCCGTGCTGCTGCCCGACAACCACTACCTGAGCACCCAGTCCGCCCTGAGCAAAGACCCCAACGAGAAGCGCGATCACATGGTCCTGCTGGAGTTCGTGACCGCCGCCGGGATCACTCTCGGCATGGACGAGCTGTACAAGTAACGCCCGCCCCACGACCCGCAGCGCCCGACCGAAAGGAGCGCACGACCCCATGCATCGGTACCTTTAAGACCAATGACTTACAAGGCAGCTGTAGATCTTAGCCACTTTCTAGAGTCGGGGCGGCCGGCCGCTTCGAGCAGACATGATAAGATACATTGATGAGTTTGGACAAACCACAACTAGAATGCAGTGAAAAAAATGCTTTATTTGTGAAATTTGTGATGCTATTGCTTTATTTGTAACCATTATAAGCTGCAATAAACAAGTTAACAACAACAATTGCATTCATTTTATGTTTCAGGTTCAGGGGGAGGTGTGGGAGGTTTTTTAAAGCAAGTAAAACCTCTACAAATGTGGTCGCTTCCTCGCTCACTGACTCGCTGCGCTCGGTCGTTCGGCTGCGGCGAGCGGTATCAGCTCACTCAAAGGCGGTAATACGGTTATCCACAGAATCAGGGGATAACGCAGGAAAGAACATG

**PspCas13b-sgRNA expression vector:**

GGTACCGATTAGTGAACGGATCTCGACGGTATCGATCACGAGACTAGCCTCGAGCGGCCGCCCCCTTCACCGAGGGCCTATTTCCCATGATTCCTTCATATTTGCATATACGATACAAGGCTGTTAGAGAGATAATTGGAATTAATTTGACTGTAAACACAAAGATATTAGTACAAAATACGTGACGTAGAAAGTAATAATTTCTTGGGTAGTTTGCAGTTTTAAAATTATGTTTTAAAATGGACTATCATATGCTTACCGTAACTTGAAAGTATTTCGATTTCTTGGCTTTATATATCTTGTGGAAAGGACGAAAC**ACCGAAGTCTTCGATATCGAAGACTTGTTG**TGGAAGGTCCAGTTTTGAGGGGCTATTACAACTTTTTTTAAAGAATTCTCGACCTCGAGACAAATGGCAGTATTCATCCACAATTTTAAAAGAAAAGGGGGGATTGGGGGGTACAGTGCAGGGGAAAGAATAGTAGACATAATAGCAACAGACATACAAACTAAAGAATTACAAAAACAAATTACAAAAATTCAAAATTTTCGGGTTTATTACAGGGACAGCAGAGATCCACTTTGGCCGCGGCTCGAGGGGGTTGGGGTTGCGCCTTTTCCAAGGCAGCCCTGGGTTTGCGCAGGGACGCGGCTGCTCTGGGCGTGGTTCCGGGAAACGCAGCGGCGCCGACCCTGGGACTCGCACATTCTTCACGTCCGTTCGCAGCGTCACCCGGATCTTCGCCGCTACCCTTGTGGGCCCCCCGGCGACGCTTCCTGCTCCGCCCCTAAGTCGGGAAGGTTCCTTGCGGTTCGCGGCGTGCCGGACGTGACAAACGGAAGCCGCACGTCTCACTAGTACCCTCGCAGACGGACAGCGCCAGGGAGCAATGGCAGCGCGCCGACCGCGATGGGCTGTGGCCAATAGCGGCTGCTCAGCAGGGCGCGCCGAGAGCAGCGGCCGGGAAGGGGCGGTGCGGGAGGCGGGGTGTGGGGCGGTAGTGTGGGCCCTGTTCCTGCCCGCGCGGTGTTCCGCATTCTGCAAGCCTCCGGAGCGCACGTCGGCAGTCGGCTCCCTCGTTGACCGAATCACCGACCTCTCTCCCCAGGGGGATCCACCATGGTGAGCAAGGGCGAGGAGCTGTTCACCGGGGTGGTGCCCATCCTGGTCGAGCTGGACGGCGACGTAAACGGCCACAAGTTCAGCGTGTCCGGCGAGGGCGAGGGCGATGCCACCTACGGCAAGCTGACCCTGAAGTTCATCTGCACCACCGGCAAGCTGCCCGTGCCCTGGCCCACCCTCGTGACCACCCTGACCTACGGCGTGCAGTGCTTCAGCCGCTACCCCGACCACATGAAGCAGCACGACTTCTTCAAGTCCGCCATGCCCGAAGGCTACGTCCAGGAGCGCACCATCTTCTTCAAGGACGACGGCAACTACAAGACCCGCGCCGAGGTGAAGTTCGAGGGCGACACCCTGGTGAACCGCATCGAGCTGAAGGGCATCGACTTCAAGGAGGACGGCAACATCCTGGGGCACAAGCTGGAGTACAACTACAACAGCCACAACGTCTATATCATGGCCGACAAGCAGAAGAACGGCATCAAGGTGAACTTCAAGATCCGCCACAACATCGAGGACGGCAGCGTGCAGCTCGCCGACCACTACCAGCAGAACACCCCCATCGGCGACGGCCCCGTGCTGCTGCCCGACAACCACTACCTGAGCACCCAGTCCGCCCTGAGCAAAGACCCCAACGAGAAGCGCGATCACATGGTCCTGCTGGAGTTCGTGACCGCCGCCGGGATCACTCTCGGCATGGACGAGCTGTACAAGTAAGTACCTTTAAGACCAATGACTTACAAGGCAGCTGTAGATCTTAGCCACTTTCTAGAGTCGGGGCGGCCGGCCGCTTCGAGCAGACATGATAAGATACATTGATGAGTTTGGACAAACCACAACTAGAATGCAGTGAAAAAAATGCTTTATTTGTGAAATTTGTGATGCTATTGCTTTATTTGTAACCATTATAAGCTGCAATAAACAAGTTAACAACAACAATTGCATTCATTTTATGTTTCAGGTTCAGGGGGAGGTGTGGGAGGTTTTTTAAAGCAAGTAAAACCTCTACAAATGTGGTCGCTTCCTCGCTCACTGACTCGCTGCGCTCGGTCGTTCGGCTGCGGCGAGCGGTATCAGCTCACTCAAAGGCGGTAATACGGTTATCCACAGAATCAGGGGATAACGCAGGAAAGAACATGTGAGCAAAAGGCCAGCAAAAGGCCAGGAACCGTAAAAAGGCCGCGTTGCTGGCGTTTTTCCATAGGCTCCGCCCCCCTGACGAGCATCACAAAAATCGACGCTCAAGTCAGAGGTGGCGAAACCCGACAGGACTATAAAGATACCAGGCGTTTCCCCCTGGAAGCTCCCTCGTGCGCTCTCCTGTTCCGACCCTGCCGCTTACCGGATACCTGTCCGCCTTTCTCCCTTCGGGAAGCGTGGCGCTTTCTCAATGCTCACGCTGTAGGTATCTCAGTTCGGTGTAGGTCGTTCGCTCCAAGCTGGGCTGTGTGCACGAACCCCCCGTTCAGCCCGACCGCTGCGCCTTATCCGGTAACTATCGTCTTGAGTCCAACCCGGTAAGACACGACTTATCGCCACTGGCAGCAGCCACTGGTAACAGGATTAGCAGAGCGAGGTATGTAGGCGGTGCTACAGAGTTCTTGAAGTGGTGGCCTAACTACGGCTACACTAGAAGGACAGTATTTGGTATCTGCGCTCTGCTGAAGCCAGTTACCTTCGGAAAAAGAGTTGGTAGCTCTTGATCCGGCAAACAAACCACCGCTGGTAGCGGTGGTTTTTTTGTTTGCAAGCAGCAGATTACGCGCAGAAAAAAAGGATCTCAAGAAGATCCTTTGATCTTTTCTACGGGGTCTGACGCTCAGTGGAACGAAAACTCACGTTAAGGGATTTTGGTCATGAGATTATCAAAAAGGATCTTCACCTAGATCCTTTTAAATTAAAAATGAAGTTTTAAATCAATCTAAAGTATATATGAGTAAACTTGGTCTGACAGTTACCAATGCTTAATCAGTGAGGCACCTATCTCAGCGATCTGTCTATTTCGTTCATCCATAGTTGCCTGACTCCCCGTCGTGTAGATAACTACGATACGGGAGGGCTTACCATCTGGCCCCAGTGCTGCAATGATACCGCGGGACCCACGCTCACCGGCTCCAGATTTATCAGCAATAAACCAGCCAGCCGGAAGGGCCGAGCGCAGAAGTGGTCCTGCAACTTTATCCGCCTCCATCCAGTCTATTAATTGTTGCCGGGAAGCTAGAGTAAGTAGTTCGCCAGTTAATAGTTTGCGCAACGTTGTTGCCATTGCTACAGGCATCGTGGTGTCACGCTCGTCGTTTGGTATGGCTTCATTCAGCTCCGGTTCCCAACGATCAAGGCGAGTTACATGATCCCCCATGTTGTGCAAAAAAGCGGTTAGCTCCTTCGGTCCTCCGATCGTTGTCAGAAGTAAGTTGGCCGCAGTGTTATCACTCATGGTTATGGCAGCACTGCATAATTCTCTTACTGTCATGCCATCCGTAAGATGCTTTTCTGTGACTGGTGAGTACTCAACCAAGTCATTCTGAGAATAGTGTATGCGGCGACCGAGTTGCTCTTGCCCGGCGTCAATACGGGATAATACCGCGCCACATAGCAGAACTTTAAAAGTGCTCATCATTGGAAAACGTTCTTCGGGGCGAAAACTCTCAAGGATCTTACCGCTGTTGAGATCCAGTTCGATGTAACCCACTCGTGCACCCAACTGATCTTCAGCATCTTTTACTTTCACCAGCGTTTCTGGGTGAGCAAAAACAGGAAGGCAAAATGCCGCAAAAAAGGGAATAAGGGCGACACGGAAATGTTGAATACTCATACTCTTCCTTTTTCAATATTATTGAAGCATTTATCAGGGTTATTGTCTCATGAGCGGATACATATTTGAATGTATTTAGAAAAATAAACAAATAGGGGTTCCGCGCACATTTCCCCGAAAAGTGCCACCTGACGCGCCCTGTAGCGGCGCATTAAGCGCGGCGGGTGTGGTGGTTACGCGCAGCGTGACCGCTACACTTGCCAGCGCCCTAGCGCCCGCTCCTTTCGCTTTCTTCCCTTCCTTTCTCGCCACGTTCGCCGGCTTTCCCCGTCAAGCTCTAAATCGGGGGCTCCCTTTAGGGTTCCGATTTAGTGCTTTACGGCACCTCGACCCCAAAAAACTTGATTAGGGTGATGGTTCACGTAGTGGGCCATCGCCCTGATAGACGGTTTTTCGCCCTTTGACGTTGGAGTCCACGTTCTTTAATAGTGGACTCTTGTTCCAAACTGGAACAACACTCAACCCTATCTCGGTCTATTCTTTTGATTTATAAGGGATTTTGCCGATTTCGGCCTATTGGTTAAAAAATGAGCTGATTTAACAAAAATTTAACGCGAATTTTAACAAAATATTAACGTTTACAATTTCCCATTCGCCATTCAGGCTGCGCAACTGTTGGGAAGGGCGATCGGTGCGGGCCTCTTCGCTATTACGCCAGCCCAAGCTACCATGATAAGTAAGTAATATTAAGGTACGGGAGGTACTTGGAGCGGCCGCAATAAAATATCTTTATTTTCATTACATCTGTGTGTTGGTTTTTTGTGTGAATCGATAGTACTAACATACGCTCTCCATCAAAACAAAACGAAACAAAACAAACTAGCAAAATAGGCTGTCCCCAGTGCAAGTGCAGGTGCCAGAACATTTCTCTATCGATA
